# Cross-species identification of genomic drivers of squamous cell carcinoma development across preneoplastic intermediates

**DOI:** 10.1038/ncomms12601

**Published:** 2016-08-30

**Authors:** Vida Chitsazzadeh, Cristian Coarfa, Jennifer A. Drummond, Tri Nguyen, Aaron Joseph, Suneel Chilukuri, Elizabeth Charpiot, Charles H. Adelmann, Grace Ching, Tran N. Nguyen, Courtney Nicholas, Valencia D. Thomas, Michael Migden, Deborah MacFarlane, Erika Thompson, Jianjun Shen, Yoko Takata, Kayla McNiece, Maxim A. Polansky, Hussein A. Abbas, Kimal Rajapakshe, Adam Gower, Avrum Spira, Kyle R. Covington, Weimin Xiao, Preethi Gunaratne, Curtis Pickering, Mitchell Frederick, Jeffrey N. Myers, Li Shen, Hui Yao, Xiaoping Su, Ronald P. Rapini, David A. Wheeler, Ernest T. Hawk, Elsa R. Flores, Kenneth Y. Tsai

**Affiliations:** 1 Department of Translational Molecular Pathology, University of Texas MD Anderson Cancer Center Houston, Houston, Texas 77030, USA; 2 Department of Dermatology, University of Texas MD Anderson Cancer Center Houston, Houston, Texas 77030, USA; 3 Department of Molecular and Cellular Biology, Baylor College of Medicine, Houston, Texas 77030, USA; 4 Human Genome Sequencing Center, Baylor College of Medicine, Houston, Texas 77030, USA; 5 Northwest Diagnostic Clinic, Houston, Texas 77090, USA; 6 Skin and Laser Surgery Associates, Pasadena, Texas 77505, USA; 7 Bellaire Dermatology, Bellaire, Texas 77030, USA; 8 Sequencing and Microarray Facility, University of Texas MD Anderson Cancer Center Houston, Houston, Texas 77030, USA; 9 Next Generation Sequencing Facility, Smithville, University of Texas MD Anderson Cancer Center Houston, Houston, Texas 77030, USA; 10 Department of Dermatology, University of Texas Medical School at Houston, Houston, Texas 77030, USA; 11 Department of Biochemistry and Molecular Biology, University of Texas MD Anderson Cancer Center Houston, Houston, Texas 77030, USA; 12 Department of Medicine, Boston University School of Medicine, Boston, Massachusetts 02215, USA; 13 Department of Biology and Biochemistry University of Houston, Houston, Texas 77204, USA; 14 Department of Head & Neck Surgery, University of Texas MD Anderson Cancer Center Houston, Houston, Texas 77030, USA; 15 Department of Bioinformatics & Computational Biology, University of Texas MD Anderson Cancer Center Houston, Houston, Texas 77030, USA; 16 Department of Clinical Cancer Prevention, University of Texas MD Anderson Cancer Center Houston, Houston, Texas 77030, USA

## Abstract

Cutaneous squamous cell carcinoma (cuSCC) comprises 15–20% of all skin cancers, accounting for over 700,000 cases in USA annually. Most cuSCC arise in association with a distinct precancerous lesion, the actinic keratosis (AK). To identify potential targets for molecularly targeted chemoprevention, here we perform integrated cross-species genomic analysis of cuSCC development through the preneoplastic AK stage using matched human samples and a solar ultraviolet radiation-driven Hairless mouse model. We identify the major transcriptional drivers of this progression sequence, showing that the key genomic changes in cuSCC development occur in the normal skin to AK transition. Our data validate the use of this ultraviolet radiation-driven mouse cuSCC model for cross-species analysis and demonstrate that cuSCC bears deep molecular similarities to multiple carcinogen-driven SCCs from diverse sites, suggesting that cuSCC may serve as an effective, accessible model for multiple SCC types and that common treatment and prevention strategies may be feasible.

## Abstract (web summary)

Cutaneous squamous cell of the skin is a common neoplasm that frequently arises from precancerous actinic keratoses. Here, the authors carry out genomic analysis on matched sets of human lesions and compare with those in ultraviolet treated mice and identify conserved drivers of tumour development.

## Graphical abstract

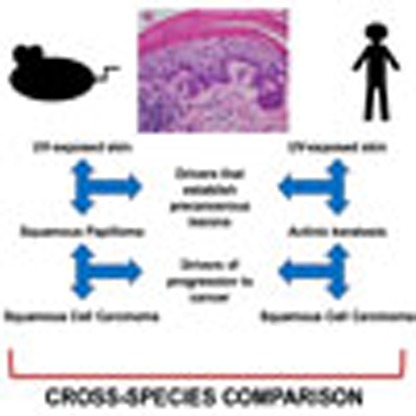

Actinic keratoses (AK) are likely the most common precancerous lesion in humans, affecting up to 5.5% of women and 13.9% of men in USA, accounting for 5.2 million outpatient visits per year at an estimated annual cost of over $1 billion^1^,^2^. AKs are scaly lesions, often readily appreciated on sun-exposed skin. Histologically, they are characterized by epidermal dysmaturation and partial thickness basal and spinous layer atypia. In time, this atypia may extend to the full thickness of the epidermis (AK3/squamous cell carcinoma in-situ) or beyond, culminating in invasive cutaneous squamous cell carcinoma (cuSCC). Ultraviolet radiation (UVR) is the main aetiological factor implicated in AK and cuSCC pathogenesis.

Approximately 0.6% of clinically diagnosed AKs are estimated to progress to cuSCC within 1 year and 2.6% are estimated to progress within 4 years^3^. Thus, the standard practice of destroying these lesions is well founded, but there is still no rationally designed way of preventing their progression, and there are still up to 700,000 cases of cuSCC in USA every year^4^. Destructive therapies are effective but management of high-risk populations such as organ transplant recipients is challenging and systemic compounds such as retinoids have substantial adverse reactions^2^,^5^. AKs are almost always treated, usually quickly and easily on an outpatient basis; however, the morbidity and economic burden of multiple treatments is high^2^,^5^. Understanding the genetic alterations that dictate AK formation and progression to cuSCC forms the molecular basis for rationally designed targeted cancer chemoprevention for an extremely common skin cancer.

To date, molecular genetic studies of AK have largely centred on known tumour suppressor genes. *TP53*, *RAS*, *CDKN2A* mutations and loss of *CDKN2A* and *p53* expression have been identified in AK^6^,^7^,^8^, as well as extensive loss-of-heterozygosity and chromosomal aberrations^9^. What dictates whether or not AKs progress to cSCC is inadequately understood as these genetic lesions are also commonly found in cuSCC. Amplifications of epidermal growth factor receptor (*EGFR*) and *c-MYC* have been identified in AK and cuSCC^10^,^11^. Loss of *INPP5A* and *CKS1B* amplification have been demonstrated in human cuSCC and smaller proportions of AKs^12^,^13^. Gene signatures that distinguish SCC from AK or irradiated skin have been identified, but they have not been refined to identify a mechanistic basis for progression^14^,^15^,^16^. Few of the multiple attempts at genome-wide analysis of AK and cuSCC^15^,^17^,^18^,^19^,^20^,^21^ have used matched histologically validated lesions from individual patients^16^ and all have employed several platforms known to have potentially high annotation error rates^22^. Given these challenges, it is not surprising that it has been difficult to identify drivers of progression when comparing tumour tissue to their normal counterparts, or when comparing unmatched samples.

In this study, we sought to identify important genetic events that drive squamous cell carcinoma (SCC) development through combined analysis of next-generation sequencing of matched patient samples with a UVR-driven mouse model to identify key pathways. Our approach minimizes the impact of inter-individual variability and annotation errors, while enabling identification of the most biologically significant pathways through cross-species analysis. We compared non-lesional, chronically UVR-exposed skin (normal skin, ‘NS' in human, ‘CHR' in mouse) to preneoplastic AK (human)/papilloma (mouse) and subsequently to cuSCC using successive pairwise comparisons as well as progression models to highlight potential targets for cancer prevention.

## Results

### Patient samples and mouse model

**Figure 1:**
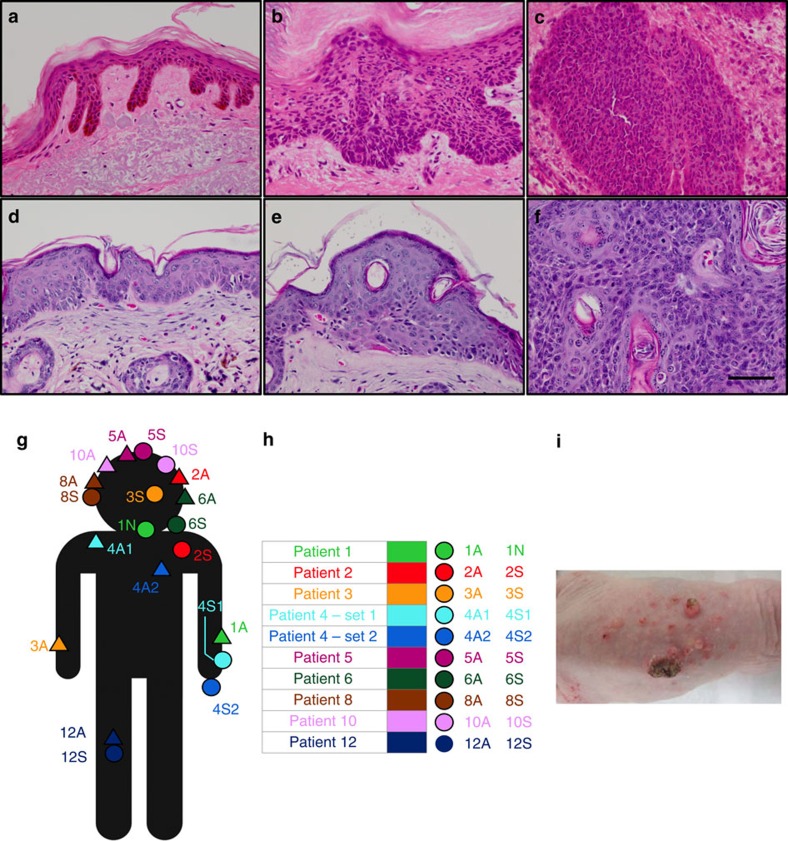
Anatomic distribution and histology of representative tissues isolated from human patients and Hairless mice. (**a**–**c**) Normal (peri-tumoural) skin, actinic keratosis and invasive cuSCC, respectively, are shown from human patients. Scale bar, 50 μm. Human samples were processed following combined RNAlater and formalin fixation, resulting in significant cytoplasmic shrinkage. (**d**–**f**) Normal (peri-tumoural) skin, papillomas and invasive cuSCC, respectively, are shown from Hairless mice. (**g**) Anatomic locations of matched samples from human patients. (**h**) Tabular list of matched samples from human patients: (S) denotes the cuSCC with adjacent NS, (N) denotes normal skin and (A) denotes the AK. For patient 1, only NS and AK were available for analysis. (**i**) Representative skin samples from Hairless mice are shown, which include smaller papillomas and a smaller number of invasive carcinomas.

A total of 27 tissue samples were isolated from nine patients who were treated for invasive cuSCC with Mohs surgery (Table 1). cuSCC tumour cores were extracted before Mohs surgery with matched samples of peri-tumoural clinically normal skin within 1 cm of the tumour removed in the course of reconstruction. For most patients, a distinct AK was also isolated, often from the same general field (Fig. 1a–c,g,h).

In parallel, we established chronically UVR-irradiated SKH-1E Hairless mice using solar simulators (Oriel) as a highly relevant model for UVR-induced human cuSCC^23^,^24^ (Fig. 1d–f,i). SKH-1E hairless mice are highly susceptible to UVR-induced skin tumours, UVR-induced immunosuppression and DNA damage^23^. Solar simulators more accurately simulate terrestrial UVR exposure than do fluorescent ultraviolet bulbs^24^. Thus our model ensures a useful platform in which we can test chemoprevention approaches. Tumours in these mice develop *p53* (ref. 25), *RAS* (ref. 26) and *CDKN2A* (ref. 27) mutations in similar proportions to those in human cuSCC, along with copy number variations that map to ones reported in human cuSCC (chromosomes 3p, 11p and 9q) (refs 28, 29, 30) Serial Analysis of Gene Expression (SAGE) mRNA gene expression data from this model, comparing UVR-induced cuSCC to NS epidermis, shows substantially similar patterns of changes to our human data including overexpression of matrix metalloproteinases and hyperproliferative keratins^31^. Importantly, these mice develop precancerous papillomas (PAP) and cuSCC following chronic low-dose UVR exposure^23^.

Six littermate female Hairless mice were chronically irradiated with 12.5 kJ m^−2^ of ultraviolet B (UVB) weekly for 100 days, and 14 days following cessation of irradiation, killed at which time, chronically irradiated skin (CHR), PAP, and cuSCC were isolated. All papillomas were grade 1 or grade 2 (not grade 3) and all cuSCC were grade 1 or grade 2 (ref. 23). All human and mouse samples were histologically validated with estimated 80% tumour cellularity for AK/PAPs and cuSCCs. The chronically UVR-exposed samples from both patients (NS) and mice (CHR) exhibited clear histologic evidence of solar damage including elastosis, fibrosis and chronic inflammation (Fig. 1).

### Mutational analysis

**Figure 2:**
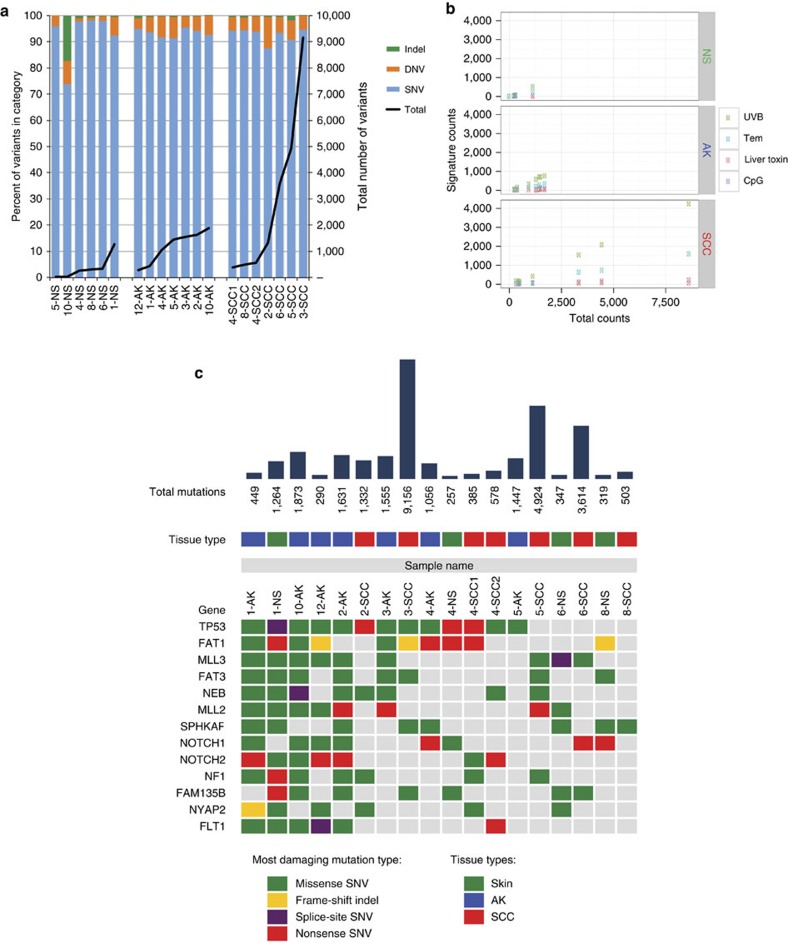
The spectrum of mutations in human samples is dominated by C toT transitions and show increasing mutation burden across cuSCC development. (**a**) cuSCC display very high mutational loads of 45.7 variants per Mb, with NS and AK samples harbouring an average of 5.8 and 18.5 variants per Mb, respectively. There are strongly dominated by single nucleotide variants. (**b**) NMF-derived orthogonal mutational profiles derived from over 6,000 human cancers confirm a strong enrichment for CpG-associated C→T transitions classically associated with UVB exposure, particularly for AK and cuSCC. For each lesion, the proportion of mutations attributable to a specific signature is plotted as a function of total mutation counts. This correlates strongly with the increasing mutational burden observed in AK and cuSCC, suggesting that the increase is attributable solely to UVB exposure. Three other profiles dominated by C→T transitions are significantly represented in the mutational data and relatively enriched in NS, including ones first described in the context of temozolamide exposure (Tem), one attributed to liver toxins, and one associated with CpG sites. These latter two signatures likely reflect background mutational processes in this context. (**c**) SMG from this cohort, match those identified previously studied cohorts of cuSCC.

Exome sequencing (Illumina Hi-Seq) was performed on a subset (Table 1) of collected samples with an average coverage of 135 × ±22 (mean±s.d.). The mutational load varied widely across our cohort of well-differentiated primary cuSCC, averaging 2,927 somatic variants (range 385–9,156) or 45.7 variants per Mb (Fig. 2a), which is congruent with previously reported results of about 50 mutations per Mb for cuSCC^32^,^33^,^34^,^35^, keeping in mind that some AKs and cuSCCs were referenced to UVR-exposed peri-tumoural NS and not germline (Table 1). AKs had substantially fewer variants, with an average of 1,186 variants (range 290–1,873) or 18.5 per Mb.

To our surprise, the clinically normal, chronically UVR-exposed skin of patients harboured an average of 372 somatic variants (range 23–1,264) across the exome when referenced to germline DNA samples obtained from saliva, corresponding to an average of 5.8 variants per Mb (Fig. 2a). This indicates that the skin sustains substantial mutagenic insults in the course of chronic UVR exposure, as recently reported in UVR-exposed eyelid skin^36^. *TP53* mutations have been described before in UVR-exposed skin; however it was not known if this represented ongoing selection specifically for *TP53* mutation^37^. Hi-depth targeted sequencing of 74 genes has demonstrated an estimated five mutations per Mb in chronically UVR-exposed eyelid skin, with a strong preponderance of *NOTCH1-3, TP53* and *FGFR3* mutations suggesting positive selection for these mutations^36^.

The spectrum of mutations is very strongly dominated by transitions between cytosine and thymine, in particular from cytosine to thymine (C→T) (Fig. 2b and Supplementary Figs 1,2a,b). The proportion of dinucleotide variants that are CC→TT is 90% for both AK and cuSCC and 84% for NS (Supplementary Fig. 1), also highly reflective of UVB exposure. Given the relative statistical rarity of CpG islands across the human genome, the high proportion of C→T transitions at CpG sites reflects the enhanced susceptibility of methylated CpG to deamination and to photoproduct formation^38^.

By using non-negative matrix factorization (NMF) -based spectral deconvolution, 21 mutation signatures were derived from over 6,000 specimens across 32 cancer types profiled in TCGA^39^. Three signatures predominated in our samples, which were strongly enriched for C→T transitions (Fig. 2b and Supplementary Fig. 2a,b). AK and cuSCC are clearly driven by UVR exposure, with substantial enrichment for the classic UVB C→T transition signature at dipyrimidines^38^ in a manner that correlated with increasing mutational load (Fig. 2b). NS samples had more evenly represented mutation signatures, including those associated with liver toxin exposure, temozolamide (Tem) exposure and CpG sites (Fig. 2b and Supplementary Fig. 2a,b).

**Figure 3:**
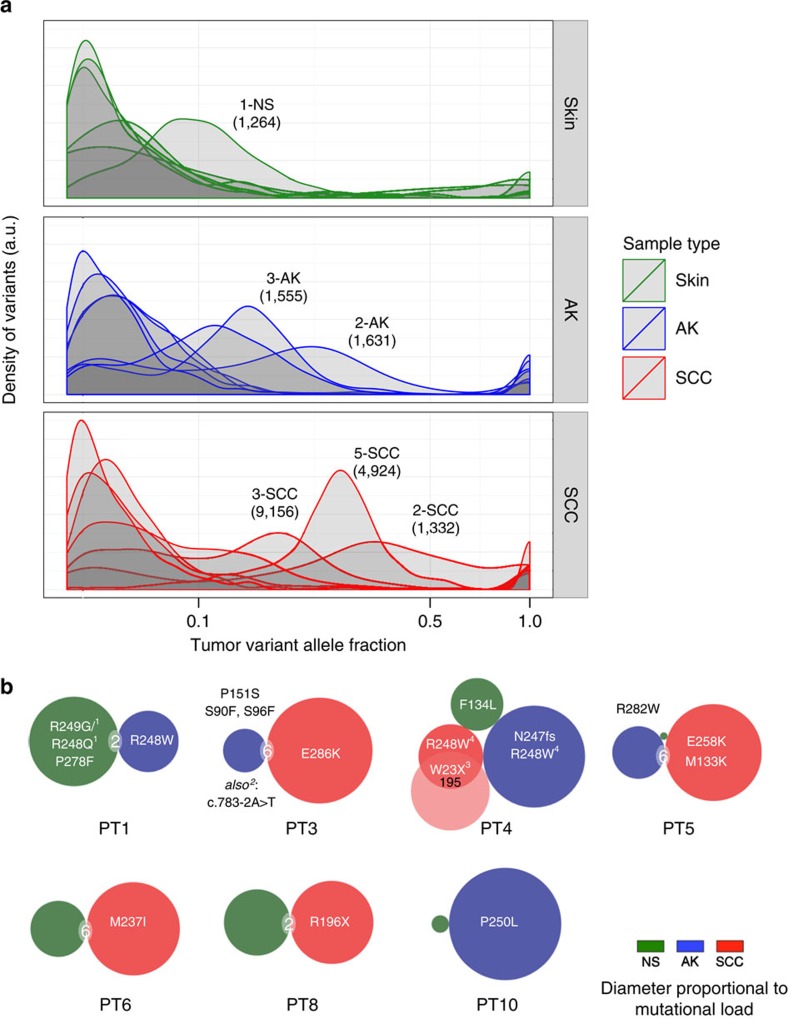
Significant mutational heterogeneity and overlap exists between AK and cuSCC. (**a**) Histograms of variant allele frequencies in NS, AK and cuSCC, show that NS have a large number of low-frequency variants. AK and cuSCC have a more heterogeneous distribution of variant frequencies, with higher-frequency variants, indicative of a general towards the emergence of dominant clones. Outliers with high frequencies of mutations in the NS and AK groups (labelled by patient #) are annotated with mutation frequencies in parentheses to show how these frequencies correlate with increasing monoclonality. (**b**) Point mutations in *TP53* compared with overall mutation counts and site-specific ovelaps. Specific mutations are indicated in text, size of the circle indicates the total number of mutations of that sample, with overlaps >1 shown between lesions. R248 is the only amino acid changed in multiple samples within patients 1, 4. Notes regarding specific findings: 1: complex variant at R248/9 hotspot; 2: splice-site variant, 3: appears in both SCC-1 and SCC-2, 4: base change differs between AK and SCC-1 (c.GG741AA in AK, and c.C742T in SCC-1).

To identify significantly mutated genes (SMG), we identified those that were recurrently mutated in at least seven pairings and that were either previously implicated in cuSCC or have COSMIC frequencies over 400 (Fig. 2c and Supplementary Data 1). These included genes found to be mutated in metastatic and aggressive cuSCC (Fig. 2c)^32^,^33^,^34^,^35^, most prominently, *TP53*, *NOTCH1-2*, *FAT1* and *MLL2*. We also identified a rare *KNSTRN* missense mutation (resulting in p.P28S) in only two pairings (one cuSCC, AK), which appears to be within the same functional domain as a previously reported hotspot p.S25F (ref. 40). Importantly, AKs not only have mutations in all of the known SMGs, but AKs have the ‘greatest' proportion of SMGs represented. This is consistent not only with the notion that AKs have acquired the mutational events necessary for cuSCC formation, but that AKs may harbour multiple clones that have the capacity to ultimately give rise to cuSCC (Fig. 3a). There is significant overlap in these variant allele frequency profiles, as all lesion types span a continuum of distributions with a trend of increasing mutational load reflecting increasing monoclonality in NS and AK samples (Fig. 3a). Of note, the NS sample (patient 1) with high variant allele frequencies, has evidence for three *TP53* mutations (Fig. 3b) highlighting how significant mutational loads can be acquired even in the absence of evident dysplasia. Given that mutations exist in hundreds of clones within UVR-exposed skin^36^ (Fig. 2a), our data suggest that dominant clones may be emerging in AK and cuSCC, particularly in the latter (Fig. 3a and Supplementary Fig. 3), although this sample size was limited.

To assess whether a pattern of mutationally driven progression could be ascertained, we then probed whether mutations overlapped between the three groups of samples. Globally, the number of site-specific mutational overlaps between any two sample classes was extremely low and not likely to be significantly different from chance, though the AK to cuSCC comparison had the majority, averaging 3.4 overlaps versus NS to AK (0.75) and NS to cuSCC (1.8) (Table 2). The most significant degree of site-specific mutational overlap occurred between two cuSCCs from patient 4 (Table 2), which were in close physical proximity (Fig. 1g; forearm versus hand), strongly suggesting that these physically distinct tumours arose at least in part from a common clone. When viewed within patients, functionally significant genes were mutated in multiple samples, including *TP53* (four patients), *FAT1* (three patients) and *MLL3* (three patients) (Supplementary Fig. 4 and Supplementary Table 1). Overlaps in AK-cuSCC were the most common even among these genes, suggesting that they are specifically targeted in the development of cuSCC.

An illustrative example is provided by *TP53*, in which multiple mutations were present (Fig. 3b). When placed in the context of overall mutational loads and site-specific overlap (Fig. 3b), it is evident that the degree of overlap did not closely correlate with physical proximity (Fig. 1g—patients 1, 3 and 4 with distant AK versus NS/cuSCC and patients 5, 6, 8 and 10 with nearby AK versus NS/cuSCC). The two cuSCCs from patient 4, which shared the greatest overlap (195), shared a *TP53*^*W23X*^ mutant (Fig. 3b). Among all the human tumour samples sequenced, only amino acid R248, which is a mutational hotspot in cuSCC^41^, was multiply altered within two patients (patients 1 and 4). These findings are consistent with the concept that mutations that inactivate tumour suppressor genes are often distributed across the entire coding region and that there has not been a strongly dominant oncogenic mutation identified in cuSCC.

### Transcriptomic analysis

**Figure 4:**
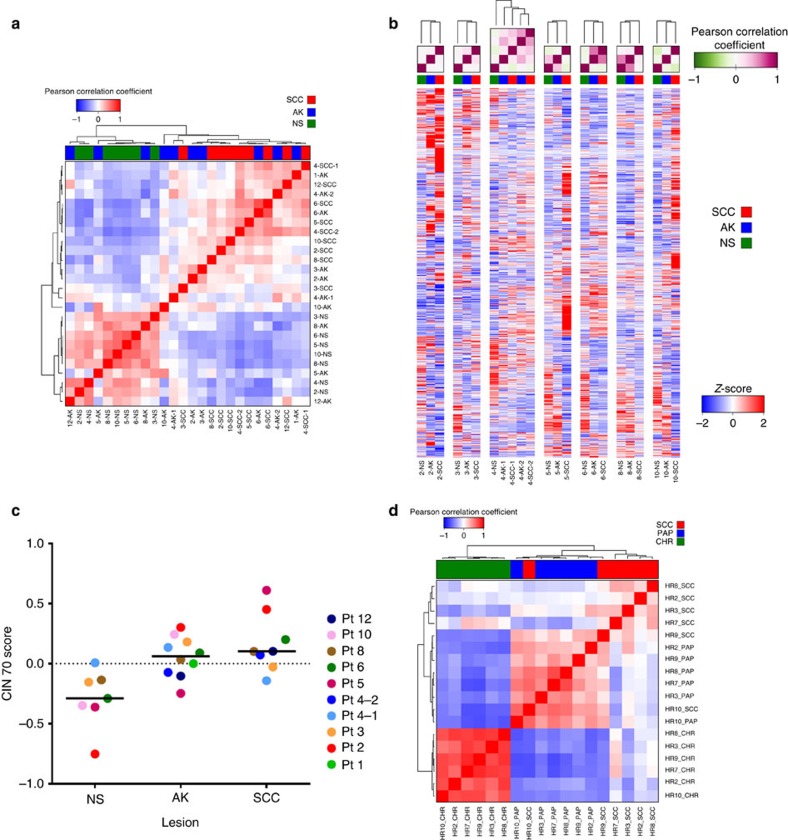
mRNA profiling across AK/papilloma and cuSCC development. (**a**) Correlation matrix of mRNAs differentially expressed in at least one signature in human samples shows that AKs span the spectrum of NS to cuSCC samples. (**b**) Unsupervised clustering of all genes across patient samples with complete sets (all three lesion types) demonstrates that in 6/8 sets, AK more closely resemble cuSCC. The Pearson correlation matrix is shown on top with the underlying heat map shown below. (**c**) A 70-gene signature of chromosomal instability derived from human cancers is highly enriched in AK and cuSCC to a similar degree, but not NS. (**d**) Correlation matrix of mRNAs differentially expressed in at least one signature in mouse samples demonstrates that PAPs much more closely resemble cuSCC than CHR.

RNA and miRNA-seq were performed on the Illumina Hi-Seq platform to yield an average of 64 million and 6.1 million reads, respectively. No significantly expressed fusion or viral transcripts were detected. A correlation matrix using mRNAs differentially expressed in at least one pairwise comparison demonstrated a clear distinction between NS and cuSCC with AKs interspersed across the spectrum (Fig. 4a), a pattern also observed in the corresponding gene-sample heatmap and principal component analysis (PCA) plots across all genes (Supplementary Figs. 5a,b and Supplementary Data 2). This shows that even given uniform histological criteria, there is a spectrum of AK that transcriptomically resembles UVR-exposed NS versus some that resemble cuSCC. Global unsupervised clustering of all expressed genes revealed that across all patient samples, six out of the eight complete sets (with tissue from all three lesion types) show that AK segregate with cuSCC by Pearson correlation, a pattern substantiated by the corresponding gene expression heatmaps (Fig. 4b). AK and cuSCC are both significantly enriched to a similar degree over peri-tumoural NS for a 70-gene chromosomal instability signature derived from multiple cancers (Fig. 4c), again reinforcing the idea that AKs have acquired key genomic features of invasive cuSCC^42^.

RNA-seq results performed on six sets of samples from Hairless mice corroborated this concept even more strongly, even though they are an outbred strain. Here, the correlation matrix of differentially expressed genes in at least one pairwise comparison clearly delineates CHR away from PAP and cuSCC (Fig. 4d), a pattern also observed in the corresponding gene-sample heatmap and PCA plots across all genes (Supplementary Figs. 5a,c and Supplementary Data 3).

**Figure 5:**
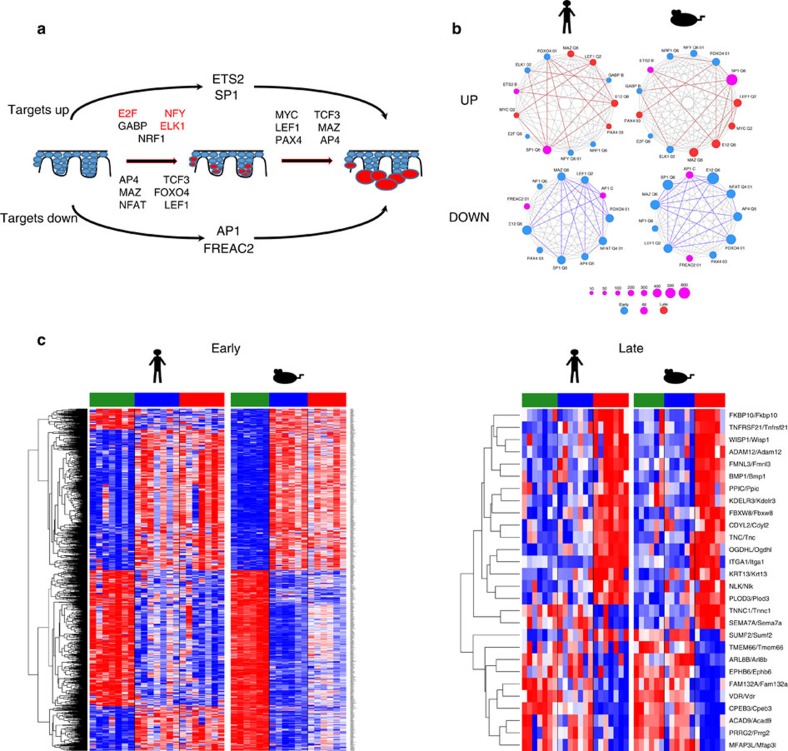
Cross-species transcription factor motif analysis reveals major drivers of cuSCC development. (**a**) Global view of transcription factors with target genes enriched across the entire NS/CHR to AK/PAP to cuSCC progression sequence. Directionality reflects the significant upregulation (above the line) or downregulation (below the line) of predicted targets of the listed transcription factors. Some factors have targets that are enriched in opposite directions across distinct transitions. The transcription factors highlighted in red were identified in both TRANSFAC and LME-based analyses. (**b**) Network analysis demonstrates that core transcriptional drivers are highly interconnected in both human (left) and mouse (right). The bolded lines delineate connections that are significant by Fisher exact test (*P*<10^−4^). (**c**) The LME model of mRNA expression changes across cuSCC development in both species demonstrates that the vast majority of significant gene expression changes occur in the early transition from NS/CHR to AK/PAP.

Next we sought to identify transcription factors that could be responsible for the gene expression changes observed in the course of AK and cuSCC development. Using TRANSFAC-based motif analysis, we identified significantly overrepresented motifs and their associated transcription factors for all pairwise comparisons in both human and mice. Although this method is not unbiased and does not definitively identify biochemical mechanisms of regulation, we surmised that cross-species analysis would enable us to identify significant drivers. First, significant candidate factors were ranked by their enrichment scores (Supplementary Data 4) and selected only if their targets were enriched in genes sets that changed in the same direction for both species in any one of the adjacent pairwise comparisons: NS/CHR to AK/PAP or AK/PAP to cuSCC. These changes also had to be significantly enriched in the NS/CHR to cuSCC comparison. This analysis yielded a total of 17 transcription factors, 11 of which were significant early, and six of which were significant late (Fig. 5a). Four factors were globally important across all pairwise comparisons: ETS2, SP1, FREAC2 (FOXF2) and AP1. Gene interaction networks assembled from the overlap of genes predicted to be regulated by multiple transcription factors reveal that these transcription factors are highly interconnected, potentially co-regulating up to several hundred genes in concert (Fig. 5b). Though these interactions must be functionally validated in detail, our data suggest that a small core of transcriptional regulators drive cuSCC development.

Importantly, some transcription factors, such as ETS2, showed largely unidirectional target modulation, but others such as TCF3 and LEF1 had significant target modulation in opposite directions, reflecting transition-specific changes (Supplementary Fig. 6a–i). Profiles of the four globally important transcription factors showed significant upregulation of ETS2 and SP1 and downregulation of FREAC2 (FOXF2) and AP1 targets across the entire progression sequence (Fig. 5a and Supplementary Data 4). ETS2 (Supplementary Fig. 6a) is pro-oncogenic in multiple other contexts, and is downstream of the ERK MAP kinase signalling module^43^, which is known to be important in sporadic and BRAF-inhibitor-induced cuSCC development^44^,^45^. The global upregulation of SP1 targets (Supplementary Fig. 6b) may reflect its ability to partner with a number of transcription factors in cancer^46^.

As expected, β-catenin/Wnt signalling plays an important role in cuSCC pathogenesis and this is reflected in the importance in this analysis of both TCF3 and LEF1 activity. TCF3 can function as a transcriptional repressor and activator and has been implicated in skin homoeostasis and wound healing^47^. Our data suggest an important role in cuSCC development as well (Supplementary Fig. 6c). LEF1 appears to be activated across the spectrum of samples, with targets significantly downregulated early and upregulated late (Supplementary Fig. 6d), and is an established effector of β-catenin/Wnt signaling^48^. The downregulation of NFAT targets early (Supplementary Fig. 6e) may reflect an inhibition of keratinocyte differentiation programs that may be modulated by NOTCH signalling and further compromise *TP53*-dependent tumour suppression^49^.

Similarly, the predicted downregulation of AP1 target genes across the continuum of cuSCC development (Supplementary Fig. 6f) suggests a compromise of normal epidermal differentiation^50^. FREAC2 (FOXF2) targets are downregulated across the development sequence (Supplementary Fig. 6g), and although it has not been specifically implicated in skin cancer, downregulation of its expression promotes epithelial–mesenchymal transition in basal breast cancer^51^.

Consistent with our global transcriptomic data, most of the transcriptional drivers are predicted to act early in the NS/CHR to AK/PAP transition, including NFY, E2F and ELK1, with some, including MYC activation, occurring late (Fig. 5a and Supplementary Fig. 6h,i). Both E2F and MYC have been implicated in cuSCC development^44^. Although using individual pairwise analysis suggested key transcriptional regulators, we also used a linear mixed effects (LME) model previously employed to identify differentially expressed genes in matched normal, premalignant and tumour samples from patients with lung SCC (LUSC)^52^. Following cross-species overlap of mouse and human data, this model also clearly demonstrated that the majority of changes occur in the earliest transition from NS to AK/PAP versus the subsequent transition to cuSCC (Fig. 5c and Supplementary Data 5).

In GATHER-based analysis of TRANSFAC motifs within concordantly differentially expressed genes^53^ by this analysis, E2F and NFY (Supplementary Fig. 6h,i and Supplementary Data 6) were again highlighted in the early stage transition from NS to AK/PAP in both mouse and human. ELK1, which is likewise regulated by the ERK MAPK pathway, was also significant by both analyses (Fig. 5a and Supplementary Data 6). No enriched transcription factor signatures were identified for the late-stage AK/PAP to cuSCC transition using the LME model.

On the basis of these sets of differentially expressed genes, Ingenuity Pathway Analysis of both human and mouse data was performed individually (Supplementary Table 2) and identified strongly overlapping key pathways including cell cycle progression, mitotic roles of polo-like kinase and DNA damage checkpoint functions, as well as upstream regulators E2F1, E2F4, CDK4, TP53, RABL6 and ERBB2, the latter two of which may be novel targets for intervention.

The transcriptional networks identified in both the pairwise analyses and the progression model analysis implicate pathways important in cuSCC development, mostly early in the NS/CHR to AK/PAP transition, notably E2F, ELK1 and NFY. ERK signalling through ETS2, β-catenin signalling through TCF3 and LEF1, and pathways regulated by NFAT and AP1 that likely impinge upon keratinocyte differentiation, were implicated globally across the entire development sequence (Fig. 5).

### MicroRNA sequencing and integrated analysis

**Figure 6:**
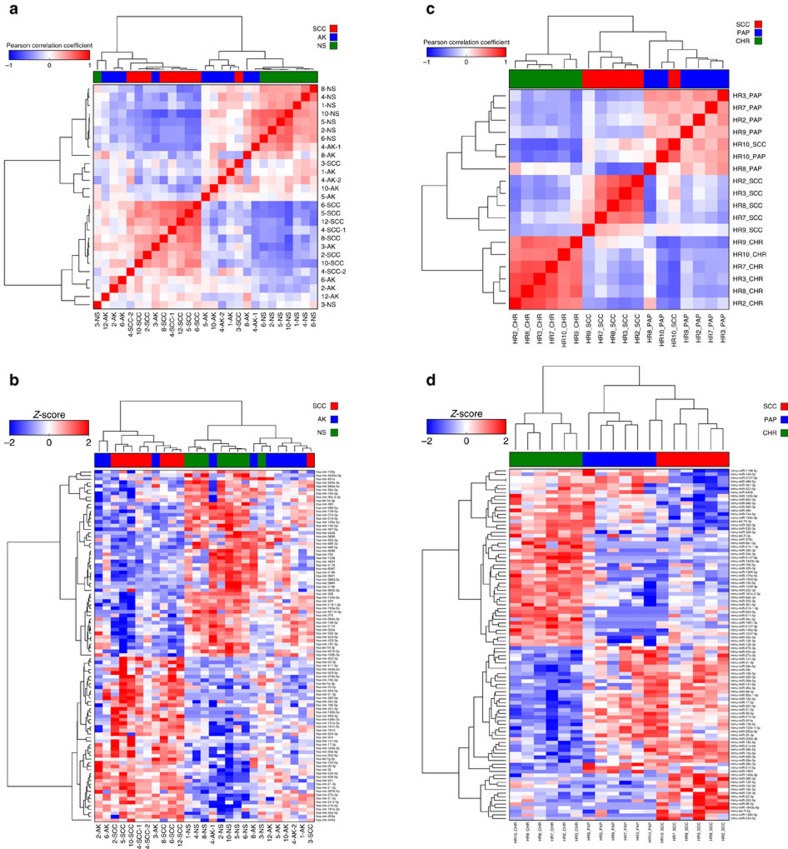
microRNA profiling across AK/papilloma and cuSCC development. (**a**) Correlation matrix of microRNAs differentially expressed in at least one signature in human samples shows that significantly improved discrimination between three sample types is achieved as compared with mRNA profiles. (**b**) Using only microRNAs differentially expressed in at least two out of three pairwise comparisons (*P*<0.05), robust discrimination is achieved between NS and cuSCC with most AKs occupying an intermediate expression pattern. (**c**) Hierarchical clustering of microRNAs differentially expressed in at least one signature in mouse samples shows distinct patterns among the three sample types as compared with mRNA profiles. (**d**) Using only microRNAs differentially expressed in at least two of three pairwise comparisons (*P*<0.05), CHR and cuSCC are very strongly segregated with an intermediate group dominated by PAP.

Relative to mRNA, clustering of microRNAs differentially expressed in at least one pairwise comparison among the matched human samples showed a better ability to distinguish the three sample classes (Fig. 6a). When recurrent statistically significant changes occurring in at least two out of three pairwise comparisons are used, it is clear that AKs now define a pattern of microRNA expression that is intermediate between NS and cuSCC, with improved discrimination between sample types (Fig. 6b, Supplementary Data 7 and Supplementary Fig. 7).

Whereas unsupervised clustering of mRNA expression in the Hairless mouse model failed to distinguish papillomas from cuSCC (Fig. 4d), unsupervised clustering of microRNA expression more clearly separates all three sample types (Fig. 6c,d, Supplementary Data 8 and Supplementary Fig. 7), suggesting that microRNAs have more discriminatory power as compared with mRNA in segregating the sample types.

**Figure 7:**
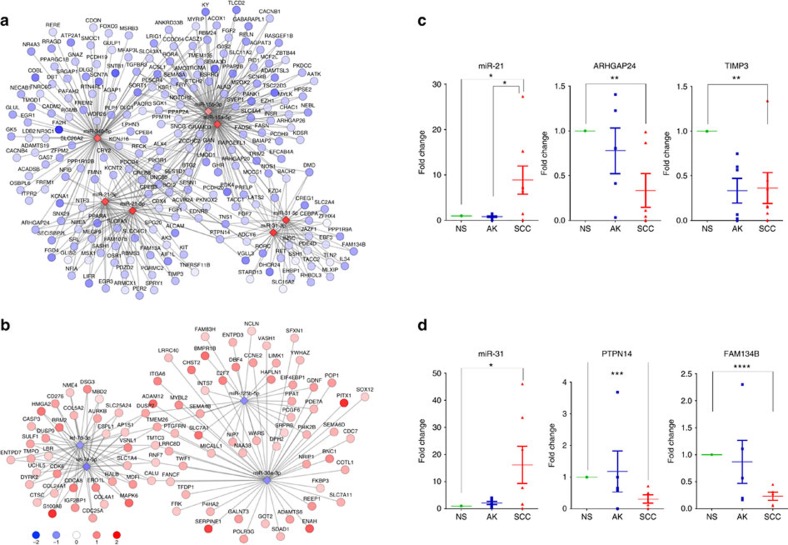
Functional pair analysis identifies highly interconnected microRNA/mRNA regulatory networks conserved across species. This occurs for both (**a**) upregulated microRNAs/downregulated target mRNAs and for (**b**) downregulated microRNAs/upregulated target mRNAs. The figures only show those microRNAs with *q*<10^−8^, log_2_-fold change of >1.15, conserved between species. Validation of select microRNA/mRNA pairs using a distinct set of human matched samples demonstrate robustness of the findings. (**c**) miR-21 is upregulated between NS and cuSCC, whereas predicted targets ARHGAP24 and TIMP3 are downregulated. (**d**) miR-31 is upregulated between NS and cuSCC, whereas predicted targets PTPN14 and FAM134B are downregulated.

Given that microRNAs can negatively regulate their target mRNA expression by Watson-Crick pairing over a 7–8 nucleotide seed region, we performed functional pair analysis by identifying miRNA–mRNA pairs predicted to be linked by a seed sequence in the 3′UTR of the mRNA and that were significantly anti-correlated in expression changes^54^. Functional pairs in both species were ranked by all possible pairwise comparisons in which they were significantly differentially expressed (rather than by stage), and an integrated network map was generated to identify several key microRNAs with multiply targeted mRNAs (Fig. 7a,b).

The microRNAs significantly upregulated in this cross-species analysis included miRs-15a/b, 17, 20a, 21, 31, 200a and 340b (Fig. 7a), and those significantly downregulated included let-7 family members and miRs-30a and 125b (Fig. 7b). Some of these microRNAs are confirmed in our data to be significant in cuSCC. miR-21 and miR-31 have been shown to be upregulated in a number of cancers including cuSCC^55^,^56^,^57^, although the identity of the most relevant mRNA targets is not known. In many instances, functionally paired mRNAs were predicted to be targeted by multiple microRNAs.

We validated the expression levels of miR-21 and miR-31 and of selected predicted target genes in additional sets (*n*=21) of matched human samples of NS, AK, and cuSCC (Fig. 7c,d and Supplementary Data 9). miR-21 was substantially upregulated by 8.9±3.1-fold (mean±s.e.m.; *n*=8 sets) across the progression sequence, with respective downregulation of the predicted targets ARHGAP24 (2.9±0.6-fold; mean±s.e.m.; *n*=6 sets) and TIMP3 (3.0±0.4-fold; mean±s.e.m.; *n*=6 sets) (Fig. 7c). ARHGAP24 is a RAC1 GAP (ref. 58), and TIMP3 suppresses metalloproteinase function, both of which are consistent with tumour suppressive functions. miR-31 was also upregulated (16.2±6.8-fold; mean±s.e.m.; *n*=7 sets) across cuSCC development and predicted to downregulate PTPN14 (3.2±0.4-fold; mean±s.e.m.; *n*=5 sets), a phosphatase which downregulates YAP signalling^59^ (Fig. 7d). In addition, FAM134B, a putative tumour suppressor^60^ was identified as a potential target of miR-31 (down 4.3±1.4-fold; mean±s.e.m.; *n*=5 sets; Fig. 7d). The let-7 family of microRNAs is prominently represented among downregulated microRNAs, consistent with a tumour suppressor role, and one of the predicted targets, HMG2A, was substantially upregulated in a validation cohort by 45.4±14.5-fold (mean±s.e.m.; *n*=4 sets). These specific pairs need to be functionally validated to provide mechanistic links, but these data show that the cross-species functional pair analysis is robust.

### Relationship of cuSCC to other SCC

Many types of SCC arising in diverse sites, such as lung, oesophagus, bladder, cervix and head and neck SCC (HNSCC) have now been genomically profiled, in addition to exome sequencing of cutaneous SCC^32^,^33^,^34^,^35^. These combined efforts have collectively identified common pathway alterations for many SCCs. *TP53* mutations occur at over 70% frequency in all SCCs; *NOTCH* family genes are mutated in over 70% of cuSCC^32^,^33^, 10–20% of HNSCC^61^,^62^,^63^, 13% of LUSC^64^ and 10% of oesophageal SCC (ESCA SCC)^65^; and SOX2 amplification is a common lineage-specific driver of SCC^66^.

**Figure 8:**
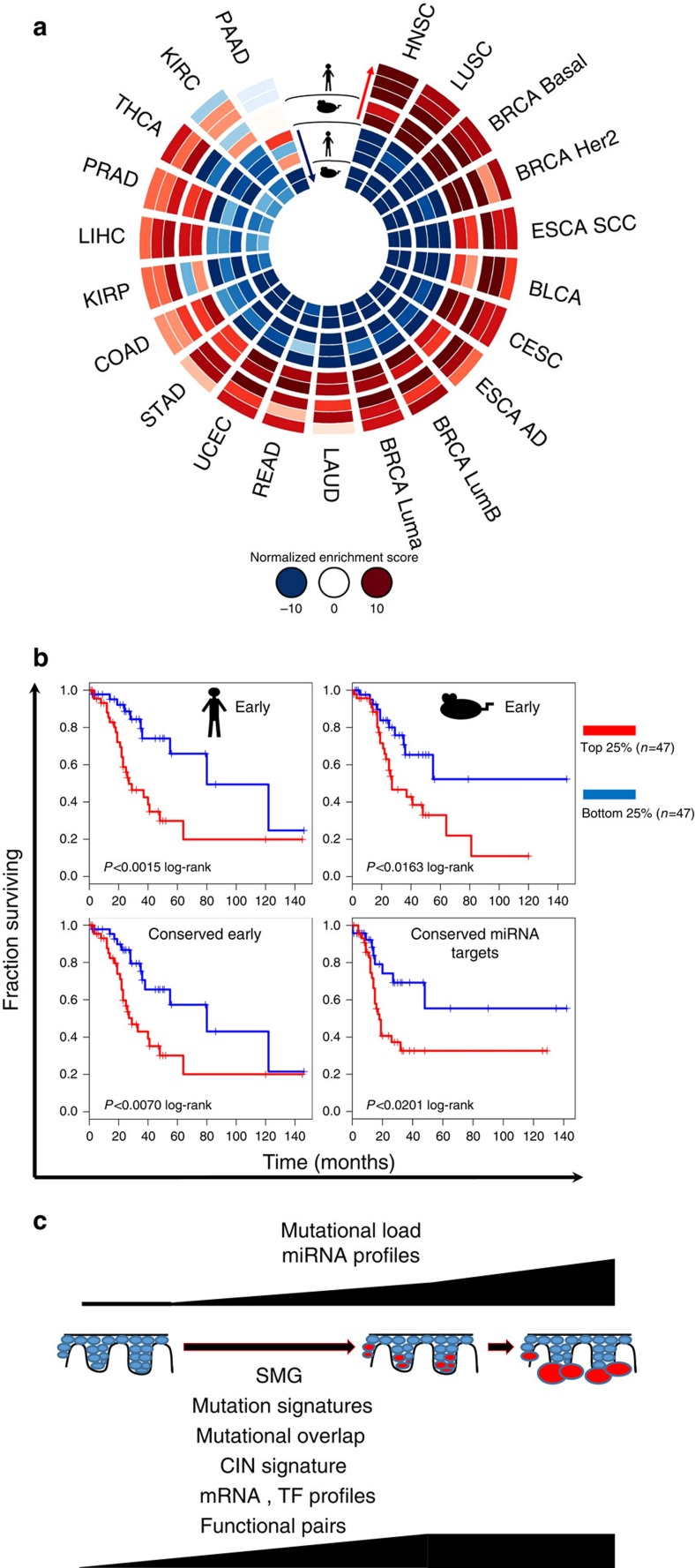
cuSCC is molecularly related to carcinogen-driven SCCs of multiple sites. (**a**) GSEA analysis of all significant pairwise comparisons in both mouse (CHR versus PAP, CHR versus cuSCC) and human (NS versus AK, AK versus cuSCC, NS versus cuSCC) represented as a CIRCOS plot. For all cancers profiled in the TCGA, normalized enrichment scores for each signature were determined and cancer types ranked by descending order (clockwise) of the sum of squares of all the scores with a penalty. By this measure, cuSCC is most closely related to HNSC, LUSC, basal and HER2+ subtypes of breast cancer (BRCA) and ESCA SCC. (**b**) Given that HNSC is most closely related to cuSCC by this measure, we show that cuSCC signatures can predict outcome (overall survival) in HNSC with *TP53* mutation, used here as a proxy for identifying tumours that do not express high-risk HPV. The cross-species early signatures derived from the linear mixed effects model and the cross-species microRNA functional analysis all predict survival in HNSCC for the top and bottom 25% of outcomes with statistical significance. Multiple hypothesis testing was performed and all of the plots shown are significant with the stated *P*-values and false discovery rate-adjusted *q*-values of <0.1 (*q*=0.021 human early, 0.070 mouse early, 0.049 conserved early and 0.070 conserved miRNA targets). (**c**) Taken together, our data show that AKs have acquired many of the properties of cuSCC as assessed by SMG, mutational overlap, mutational signatures, chromosomal instability signature, mRNA and transcription factor profiles and functional pair analysis, although overall mutational load and unsupervised microRNA clustering do enable separation of the three sample types.

To test the hypothesis that mRNA expression profiles would reveal molecular commonalities between all SCC, we profiled our NS/cuSCC signature against cancers in the TCGA using gene set enrichment analysis (GSEA). Furthermore, we surmised that there would be major differences between carcinogen-driven versus virally driven SCC, as observed in HNSCC^61^,^62^,^63^. We found that cuSCC is most similar to HNSCC, followed by LUSC, basal (triple-negative) and HER2 breast cancer and ESCA SCC, but not closely related to cervical SCC, which is overwhelmingly human papillomavirus (HPV) -driven (Fig. 8a and Supplementary Data 10). This establishes transcriptomic evidence that deep molecular commonalities exist between carcinogen-driven SCCs of multiple tissue sites, a concept supported by the mutational data, and suggests that common molecular strategies for prevention and treatment of multiple types of SCCs may exist.

Given that HNSCC is consistently the most closely related tumour to cuSCC, we asked whether cuSCC-derived signatures from our cohort of well-differentiated tumours could also be used to predict outcomes in carcinogen-driven (non-HPV) HNSCCs, which have *TP53* mutations and high mutational loads^63^. When we restricted the cuSCC signatures to early genes identified in the LME progression model for either human or mouse independently (Fig. 5c), both signatures were significantly predictive of overall survival (Fig. 8b—top row). Similarly, when we used only the cross-species intersection of these two signatures of early genes, this was again significantly predictive of overall survival (Fig. 8b—bottom left, Supplementary Fig. 8). Most importantly, a 309-gene signature derived solely from the cross-species microRNA functional pair analysis (Fig. 7a,b) had significant predictive power for overall survival (Fig. 8b—bottom right), showing that these microRNA target genes likely regulate not only important processes in cuSCC development but also drivers of disease outcome in HNSCC.

## Discussion

Our analysis is the first comprehensive characterization of genomic changes that drive the development of cuSCC through its preneoplastic intermediate, the AK, employing the combination of matched human patient samples, next-generation sequencing, and cross-species analysis. Despite the clear clinical and histological distinctions between cuSCC, AK and perilesional UVR-damaged skin, AK/PAP are most closely related to cuSCC, by many measures including mRNA expression (unsupervised clustering and LME model), transcription factor motif analysis, mutational signatures and overlap, chromosomal instability signature expression, and microRNA–mRNA functional pair analysis (Fig. 8c).

Our data confirm the high mutational burden of these skin cancers^32^,^33^,^34^,^35^. Importantly, high mutational loads have recently been described in chronically UVR-exposed blepharoplasty samples^36^, and, for the first time, we show the large degree of mosaicism present across the entire exome in non-lesional UVR-exposed peri-tumoural skin, with quantitatively similar mutational loads (Fig. 2). The SMGs identified, including *TP53*, *NOTCH1-2*, *FAT1* and *MLL2* are ones likely to be important in cuSCC pathogenesis (Fig. 2c)^32^,^33^,^34^,^35^. Conversely, one of the most frequently mutated genes previously identified in sun-exposed skin, *FGFR3*, was not found to be mutated, suggesting that this genetic lesion, frequently found in seborrheic keratoses, may be specifically critical for benign keratoses and not non-melanoma skin cancers^36^. Our findings are consistent with the notion that tumour suppressor genes, which represent the largest class of cancer genes known to be recurrently targeted in cuSCC, can often be inactivated by mutational insults spread across their entire coding regions.

The overwhelming preponderance of epidemiological, clinical and biological data suggest that UVR exposure is the main driver of sporadic cuSCC development. The subsequent expansion in overall mutational load, which occurs in progression to AK and cuSCC correlates with a significant enrichment for UVB-signature mutations (Fig. 2b). It is possible that this is reflective of a progressively more clonal structure across the continuum from NS to AK to cuSCC, although our sample size is small (Fig. 3a). The lower relative representation of the UVB signature in the NS samples maybe due to low sensitivity for detecting small subclones induced by UVR exposure at a given sequencing depth (Fig. 2b). It is possible that initiated clones persist longer than normal keratinocytes, thereby enabling expansion or accumulation of further UVB-mediated DNA damage, a notion consistent with the early appearance of *TP53* mutations (Figs 2c and 3b)^36^, and dramatically illustrated in the NS sample from patient 1 (Figs 2a,b and 3b). Other contributory mechanisms could include compromised DNA repair, compromised photoprotection by melanocytes, or the generation of these types of mutations in the absence of UVR exposure. Finally, the overlap in specific mutations was low (Fig. 3b, Table 2) and while this may also be due to the inability to detect small subclones particularly in the NS and AK samples, all the lesions (particularly AKs) were physically distinct. Nevertheless, the dramatic overlap in mutations between the two separate SCCs in patient 4 strongly suggests that clones of UVR-initiated keratinocytes can populate large areas of epidermis (Fig. 3b and Table 2).

SCCs arise at interfaces with the environment, thus making them susceptible to sustained carcinogenic insults. Our data support the notion that cuSCC, HNSCC, LUSC and ESCA SCC share deep molecular commonalities (Fig. 8a) at the mutational and transcriptional levels, and include deregulation of key pathways such as those driven by altered *RB1*, *TP53* and *TP63* function^32^,^34^,^35^,^63^,^64^,^65^. Therefore, for the subsets of these SCCs driven by UVR, alcohol and tobacco exposure, common molecular treatment and prevention strategies may potentially be developed and modelled on cuSCCs, which are substantially more accessible and common. The unexpected molecular similarity of cuSCC to specific subtypes of breast cancer may also highlight similar molecular vulnerabilities such as ERBB2/HER2 (Fig. 8a and Supplementary Table 2). Interestingly, there was much less similarity between cuSCC and cervical SCC (Fig. 8a). cuSCC does not appear to require HPV transcription for tumour maintenance, whereas cervical SCC is overwhelmingly driven by high-risk αHPV infection^67^.

While AKs appear already to harbour the majority of events that are retained in cuSCC, at least two alternative explanations are possible: (1) consistent mutational or transcriptomic events that separate AKs versus cuSCC could be present, and/or (2) there are distinct molecular classes of AKs with different risks of progression to cuSCC. Either of these explanations would require much larger numbers of samples to demonstrate. Nevertheless, our data show that microRNA expression distinguishes the three sample classes and may potentially serve as a basis for distinguishing different types of AKs (Fig. 6). In addition, our cross-species functional pair analysis has identified a handful of highly interconnected microRNA–mRNA networks that drive cuSCC development through preneoplastic AKs (Fig. 7), highlighting specific microRNA targets for potential intervention.

Nevertheless, given the many significant similarities between AK and cuSCC, our data suggest that non-lesional carcinogen-exposed fields of tissue may represent the most effective point of intervention for molecularly targeted chemoprevention. The development of cancer through a preneoplastic intermediate has been studied extensively in Barrett's oesophagus and oesophageal adenocarcinoma^68^,^69^. Barrett's oesophagus that evolves into adenocarcinoma can be largely indistinguishable from carcinoma, consistent with our conclusion that for a subset of patients, field-based treatment is most appropriate. AKs are typically too small to allow for repeated sampling, thus removing the possibility for longitudinal follow-up and clinical discernment between AKs that ultimately progress and those that do not. Nevertheless, it is likely that AKs are polyclonal reservoirs from which cuSCCs can arise (Fig. 3a), and it is unclear whether catastrophic genomic instability drives this late transition.

Our data substantiate the power of cross-species analysis to identify biologically important pathways in cuSCC development and suggests that the solar UVR-exposed Hairless mouse model is a useful testbed for chemopreventive and treatment modalities. Other mouse models of cuSCC have also been extensively studied, most prominently the DMBA/TPA model^70^; in this model, tumorigenesis is overwhelmingly driven by *Hras*^*Q61*^ mutations which are rarely found in UVR-driven human cuSCC and in the UVR-driven Hairless mouse model (Supplementary Data 1)^24^,^32^,^33^,^34^,^35^. The key transcriptional drivers we identified in both pairwise signatures and the LME model are important early in the NS/CHR to AK/PAP transition and include E2F, ELK1 and NFY. Globally, we identified ERK signalling through ETS2 and ELK1, β-catenin signalling through TCF3 and LEF1, and possible differentiation pathways regulated by NFAT and AP1 as potentially important drivers of cuSCC development (Fig. 5).

The significant enrichment of signatures from both patient samples and the UVR-driven Hairless model in multiple carcinogen-driven SCCs arising in diverse sites is important in that it establishes the concept that these SCCs are molecularly closely related (Fig. 8a). Furthermore, early cuSCC signatures were able to predict survival in *TP53*-mutant (non-HPV) HNSCC (Fig. 8b). This suggests that a common set of biological processes underlie the development of multiple SCC types, and that cuSCC may serve as an accurate and extremely accessible model for exploring pathogenesis and testing interventions.

## Methods

### Human tissue samples

All human tissues were studied under a MD Anderson Cancer Center IRB-approved protocol (LAB08-0750). All human tissues were obtained from patients who provided written informed consent and who had no history of immunosuppression. These samples were validated by histological analysis and processed using standard methods to yield both high-quality DNA and RNA (RIN>8.0).

### Mouse model of UVR-driven cuSCC

All mouse studies were conducted under MD Anderson Cancer Center IACUC-approved protocol (ACUF 00001396-RN00). Mice were obtained from Charles River Laboratories. To model UVR-driven cuSCC under controlled conditions, we exposed female SKH1-E Hairless mice to chronic low-dose UVR (12.5 kJ m^−2^ UVB total weekly divided in three doses M, W, F) using solar simulators (Oriel) starting at 3 months of age. In this strain, 5.0 kJ m^−2^ UVB is ∼0.5–1 mean erythemal dose^71^. These mice lack the Hairless gene, and they are highly susceptible to UVR-induced skin tumours, UVR-induced immunosuppression and DNA damage^23^. In our studies, we have used solar simulators with well-characterized spectra at 2.5–5.0 kJ m^−2^ UVB 3 days a week for a total of 12.5 kJ m^−2^ per week (275–325 nm) (ref. 71). These doses were verified by broadband UVB and ultraviolet A (UVA) measurements (ILT1700/ILT73B) in experimental conditions before this experiment. Doses of UVB and UVA averaged 12.5 and 145 kJ m^−2^ weekly, respectively. In this model of UVR-driven cuSCC development, we irradiated the mice for 100 days. Papillomas were typically observed within this time and were histologically well-differentiated. A minority of these lesions to invasive well-differentiated cuSCC.

### Preparation of RNA for illumina sequencing

Tissue specimens (50–200 mg) were homogenized with an Omni rotor stator homogenizer in TRIZOL (Invitrogen Cat # 15596018). Total RNA was extracted according to the manufacturer instructions. RNA purification was carried with Purelink RNA kit (Invitrogen Cat #12183018A). 4–10 μg of RNA per sample was submitted for 76 nt paired-end sequencing by lllumina HiSeq 2000. The same samples were submitted to the laboratory of Preethi Gunaratne, PhD (University of Houston, Biology & Biochemistry) for small RNA sequencing.

### DNA isolation and exome seq

PureLink Genomic DNA mini kits were used to extract DNA from tissue samples (Cat # K1820-01). Briefly, tissue specimens were minced and incubated overnight (55 °C) in PureLink genomic digestion buffer and proteinase K. Manufacturer's protocol was followed for purification using the spin columns. Two microgram of DNA per sample was submitted to MD Anderson DNA Analysis Facility for sequencing (Illumina HiSeq200, 76 nt PE). We used DNA Genotek ORAgene saliva collection kits and followed manufactures' collection and storage instructions (catalogue # OG-500). Genomic DNA was isolated from saliva samples using DNA Genotek prepIT·C2D (PT-C2D) extraction columns and manufacturers' protocol.

### Exome analysis

For any given patient, if their saliva samples were available, they were used as the paired control for the mutation detection. Otherwise, NS samples were used as controls. Four precapture libraries were pooled together and hybridized according to the manufacturer's protocol NimbleGen SeqCap EZ Exome Version 3. Exomes were sequenced on an Illumina HiSeq 2000 platform to an average coverage of 135X. Sequencing runs generated approximately 300–400 million successful reads on each lane of a flow cell, yielding 9–12 Gb per sample. Initial sequence analysis was performed using the HGSC Mercury analysis pipeline (https://www.hgsc.bcm.edu/software/mercury). First, the primary analysis software on the instrument produces.bcl files that are transferred off-instrument into the HGSC analysis infrastructure by the HiSeq Real-time Analysis module. Next, the vendor's primary analysis software (CASAVA) demultiplexes pooled samples and generates sequence reads and base-call confidence values (qualities). Reads are mapped to the GRCh37 Human reference genome (http://www.ncbi.nlm.nih.gov/projects/genome/assembly/grc/human/) using the Burrows-Wheeler aligner (BWA, http://bio-bwa.sourceforge.net/) and producing a BAM file. Finally, quality is recalibrated (GATK, http://www.broadinstitute.org/gatk/), and separate sequence-event BAMs are merged into a single-sample-level BAM. BAM sorting, duplicate read marking, and realignment to improve in/del discovery all occur at this step. Mutations were identified by using the HGSC Cancer Genomics pipeline which includes variant calling using Atlas-SNP, Atlas-INDEL, and PInDel on each of the BAM files and then merging the variant calls and performing allele lookups. Merged variant files were annotated using dbSNP, COSMIC and Annovar and then split into somatic and germline calls based on variant quality and segregation. Variant calls were then filtered using a cohort filtering approach and filtered variants were analysed. DNPs were collapsed with their neighbours and annotated by using Provean (provean.jcvi.org). Identification of SMGs essentially paralleled our previously established pipeline^34^.

### Non-negative matrix factorization-based mutation signatures

NMF-based spectral deconvolution was used to derive signatures based upon mutations placed in trinucleotide context. The method used is mathematically similar to one recently applied to similar cancer data sets^72^, but has the advantage of removing the partial overlap between the reported signatures that resulted in high correlations between some of them^39^. Our strategy employed non-smooth NMF, a variant, which approximates the data using the basis and coefficient matrices as above with the addition of a third smoothing matrix which serves to absorb noise within the data, thus driving the coefficient and basis matrices to increase sparseness. The resulting basis matrix generated *k*=21 signatures from a diverse set of over 6,000 cancers^39^, reducing correlations between signatures originally derived by Alexandrov *et al*.^72^ thereby increasing orthogonality. This enhanced orthogonality has the potential advantage of suggesting biological mechanisms of mutation generation with greater specificity.

### RNA-Seq analysis

RNA sequencing (Illumina Hi-Seq) yielded 30–40 million read pairs for each sample. The mRNA-seq paired-end reads were aligned to the human reference genome, GRCh37/hg19, using the MOSAIK alignment software^73^. The mRNA-Seq mouse sample reads were aligned onto the mouse genome build UCSC mm10 (NCBI 38). The overlaps between aligned reads and annotated genomic features, such as genes/exons were counted using the HTSeq software platform (http://www-huber.embl.de/users/anders/HTSeq/doc/overview.html). The counts were normalized using the scaling factor method. To perform further analysis, the normalized counts were transformed by the variance-stabilizing transformation method and were corrected for experimental batch effects. Specifically, the median expression of each batch was scaled to the same value per gene. Hierarchical clustering analysis, using the Pearson correlation coefficient as the distance metric, complete linkage, and PCA were performed using the R statistical system (https://www.r-project.org). Genes significantly differentially expressed between the NS/CHR, AK/PAP, and cuSCC stages were determined using the R package DESeq2 (ref. 74). Since multiple genes were tested simultaneously, the Benjamini-Hochberg method was used to control false discovery rate. For further integration of mRNAs and miRNAs, and detection of enriched transcription factor targets, we used a cutoff of *Q*-value<0.25 and fold-change exceeding 1.25 × .

Viral and fusion transcripts were assessed using the VirusSeq algorithm^75^ given that mapping viral integration sites is computationally similar to identifying fusion events. In brief, non-human reads were aligned to viral sequences from the Genome Information Broker for Viruses (http://gib-v.genes.nig.ac.jp/) with overall counts registered. Discordant paired-end reads that support a fusion event were clustered and the candidates reported using supporting pairs (at least four) and junction spanning reads (at least one) as the cut-offs.

TRANSFAC-based analysis (http://www.gene-regulation.com/index2.html) was performed by first identifying significantly overrepresented motifs and their associated transcription factors for all pairwise comparisons in both human and mice. First, significant candidate factors were ranked by their enrichment scores (Supplementary Data 4) and selected only if their targets were enriched in genes sets that changed in the same direction for both species in any one of the adjacent pairwise comparisons: NS/CHR to AK/PAP or AK/PAP to cuSCC. These changes also had to be significantly enriched in the NS/CHR to cuSCC comparison.

To quantify chromosomal instability (CIN), CIN70 score was calculated by summing up the normalized counts of all CIN70 genes^42^. To adjust the effects of multiple samples per patient and experimental batches, CIN70 scores were first transformed by the variance-stabilizing transformation method implemented in DESeq2 and fit into a linear model with patients and experimental batches as covariates. The residuals of the linear model were treated as adjusted CIN70 scores.

### Cross-species linear mixed effects model

This analysis was confined to human samples that had complete matched sets of lesion types (21 samples from six patients). Samples from all six mice were used. For each gene, LME models were constructed in which normalized gene expression (as above) was modelled as a function of sample type (fixed effect) while correcting for the patient or mouse source (random effect). The models were created twice, using either NS/CHR or AK/PAP as the reference group to compute coefficients, and *t* tests were performed to assign significance to each gene with respect to each coefficient in each model. From those genes for which both iterations of the model were successfully fitted to the data (numerically stable), a set of genes with nominal *P*<0.05 for the cuSCC versus NS/CHR coefficient was identified. Each of these sets was then filtered to identify genes for which the AK/PAP versus NS/CHR or the cuSCC versus AK/PAP coefficient also had a nominal *P*<0.05 and a sign matching that of the cuSCC versus NS/CHR coefficient; these genes were designated as early and late, respectively, or stepwise (both early and late). LME modelling and *t* tests were carried out using the R package v.3.1-97 ((https://www.r-project.org).

For cross-species analysis, the NCBI HomoloGene resource (version 68) (http://www.ncbi.nlm.nih.gov/homologene) was used to facilitate cross-species analysis as follows. First, HUGO identifiers were converted to Entrez Gene identifiers. Most (>95%) of the identifiers could be translated in this manner (16,155 out of 16,952 human features and 14,084 out of 14,542 mouse features); features that did not map to an Entrez Gene ID were discarded. Next, the resulting Entrez Gene identifiers were matched between species by common HomoloGene identifier, and any feature from either data set that did not map to exactly one homologue in the other data set was discarded. Finally, the LME results from the two data sets were merged by HomoloGene identifier to allow for direct comparison.

GATHER (Gene Annotation Tool to Help Explain Relationships)^53^ was used to identify TRANSFAC identifiers that were significantly overrepresented in the early (NS/CHR to AK/PAP), late (AK/PAP to cuSCC), and stepwise (both early and late) gene sets, with a Bayes Factor >3 considered significant.

### smallRNA-Seq analysis

This work was performed with collaboration with laboratory of Dr Preethi Gunaratne, PhD (University of Houston, Biology & Biochemistry). Illumina small RNA adapter sequences were trimmed from the reads, and reads of length below 10nt or ending in homopolymers of length 9 nt or above were discarded. Total usable number of reads for each sample was calculated. The reads were mapped to the miRBase^76^ reference (http://www.mirbase.org/) using BLAST; the abundance of each expressed microRNA was quantified as a fraction of the usable reads, and expressed as parts per million To reduce potential batch effects due to sample collection and preparation at different times, the ComBat normalization algorithm^77^ (http://www.bu.edu/jlab/wp-assets/ComBat/Abstract.html) was applied for the human data. We determined differentially expressed microRNAs imposing a fold-change of 1.5 × and *t*-test comparison (*P*<0.05) using the R statistical system. We employed PCA to examine sample structure; further visualization of microRNA significant in one or multiple comparisons was carried out using the R statistical system.

### Integrative mRNA-miRNA functional pair analysis

We determined enriched miRNA–mRNA pairs using the SigTerms methodology. Essentially, by applying a one-sided Fisher exact test and using TargetScan^78^ (http://www.targetscan.org/vert_71/) predicted microRNA targets, we determined the miRNAs for which the gene targets are significantly enriched (false discovery rate-adjusted *q*<0.25; fold-change>1.25 × ) in the gene signature, separately for the human specimens and the mouse samples. Finally, we determined the conserved enriched miRNAs alongside the SCC progression model, and the conserved miRNA–mRNA pairs conserved alongside the SCC progression model. Conserved enriched microRNA–mRNA pairs were visualized using the Cytoscape software (http://www.cytoscape.org/).

### Quantitative real-time PCR validation analysis

Separate cohorts of matched samples from patients consisting of NS, AK and cuSCC were processed as above. Commercially available Taqman (Life Technologies) probes were acquired for human miR-21 (000397), miR-31 (002279), PTPN14 (Hs00193643_m1), FAM134B (Hs00375273_m1), HMG2A (Hs00171569_m1), TIMP3 (Hs00165949_m1) and ARHGAP24 (Hs01097580_m1) and used in qRT-PCR based quantitation of expression in these tissues, as benchmarked to RNU6B (001093, microRNA) and 18S rRNA (Hs99999901, mRNA).

### Gene set enrichment analysis for TCGA tumour signatures

GSEA was carried out using the GSEA software package^79^ (http://software.broadinstitute.org/gsea/index.jsp) to assess the degree of similarity among the studied gene signatures. For each of the human or the mouse SCC progression transcriptome response, all genes were ranked by the fold change alongside the SCC progression model. To assess the relative association with multiple tumour progression signatures, we downloaded from the Cancer Genome Atlas (TCGA) (https://tcga-data.nci.nih.gov/tcga/) gene expression data for 19 cancer cohorts, performed quantile normalization using the R statistical analysis system, and then inferred tumour progression transcriptome signature by imposing a fold change exceeding 2 (*P*<0.05). We utilized separately the downregulated genes and the upregulated genes. Normalized Enrichment Score (NES) and adjusted *q*-values (*q*<0.25) were computed utilizing the GSEA method, based on 1,000 random permutations of the ranked genes. We visualized combined NES scores for all the TCGA tumour development gene signatures and for our human and mouse SCC progression signatures using the Circos software^80^ (http://circos.ca/).

### Survival analysis for TCGA HNSCC with *TP53* mutations

We evaluated the survival prognostic power of cuSCC progression-associated gene signature using human specimen cohorts from the Cancer Genome Atlas (TCGA) (https://tcga-data.nci.nih.gov/tcga/), specifically HNSCC which are *TP53*-mutant. We first replaced the gene expression of each gene with the *z*-score within the cohort, then we computed the sum of *z*-scores for each sample by adding the *z*-score for upregulated genes and subtracting the *z*-score from downregulated genes. Specimens were sorted according to the sum *z*-score of the respective SCC progression gene signature; association with survival (*P*<0.05, log-rank test) was evaluated by using the package Survival in the R statistical system (https://cran.r-project.org/web/packages/survival/index.html).

### Data availability

Raw RNA and small RNA sequencing data from all human and mouse samples that were used to support the findings of this study have been deposited in NCBI/GEO with SuperSeries accession code GSE84194. The exome sequencing data that support the findings of this paper may be made available upon request from the corresponding author (K.Y.T.). These exome data are not publicly available due to them containing information that could compromise research participant privacy/consent. The data used to support the pan-cancer GSEA tumour signature analysis used publicly available Cancer Genome Atlas (TCGA) gene expression data, which can be accessed through the websites: (https://tcga-data.nci.nih.gov/tcga/) (https://gdc-portal.nci.nih.gov/).

## Additional information

**How to cite this article:** Chitsazzadeh, V. *et al*. Cross-species identification of genomic drivers of squamous cell carcinoma development across preneoplastic intermediates. *Nat. Commun.* 7:12601 doi: 10.1038/ncomms12601 (2016).

## Supplementary Material

Supplementary Information Supplementary Figures 1-8 and Supplementary Tables 1-2

Supplementary Data 1 Significantly mutated genes.

Supplementary Data 2 Differentially-expressed genes in human samples.

Supplementary Data 3 Differentially-expressed genes in mouse samples.

Supplementary Data 4 GSEA results in transcription factor (TRANSFAC) analysis.

Supplementary Data 5 Linear mixed effects model results. For the homolog of each gene in each dataset, the HUGO symbol and Entrez Gene identifier are indicated, as well as whether the LME models converged (i.e., were numerically stable) for all coefficients and whether each gene was designated as early, late, stepwise, or none (blank). For each comparison, signed fold changes were computed in linear space, i.e., after anti-log2-transformation, and in a paired manner, i.e., the mean of the fold changes computed between pairs of anti-log-transformed values within each patient, and t statistics, nominal p values and FDR q values were computed for the corresponding coefficient. Batch-corrected FPKM values are shown, laid over a heatmap scaled so that red and blue indicate expression values {greater than or equal to} 2 standard deviations above and below, respectively, the mean expression within each patient or animal (white).

Supplementary Data 6 GATHER-based analysis of overrepresentation of transcription factor motifs in "early" genes in human and mouse. This file includes four separate tabs, dividing genes by direction of regulation (up- or down-regulated from NS/CHR to AK/PAP) and again by concordance or discordance between species. For each TRANSFAC motif, the nominal p value, Bayes Factor, and symbols of target genes are indicated for each species. The rows in the two "concordant" tabs are sorted in ascending order by human p value and filtered to show only those motifs for which the Bayes Factor is greater than 3 in both species. The rows in the two "discordant" tabs are sorted in descending order by maximum Bayes Factor and filtered to show only those motifs for which the maximum Bayes Factor across both species is greater than 3.

Supplementary Data 7 Differentially-expressed microRNAs in human samples. The underlying data for the correlation matrix, differentially expressed microRNAs across all samples, and individual signatures in pairwise comparisons are included as separate sheets.

Supplementary Data 8 Differentially-expressed microRNAs in mouse samples. The underlying data for the correlation matrix, differentially expressed microRNAs across all samples, and individual signatures in pairwise comparisons are included as separate sheets.

Supplementary Data 9 Significant microRNA-mRNA functional pairs.

Supplementary Data 10 GSEA analysis of cuSCC signatures across TCGA cancers

## Figures and Tables

**Table 1:** Clinico-pathological characteristics of patient cohort and sequencing performed.

**Patient**	**Sample name (NS/AK/SCC)**	**Gender**	**Location**	**Age (years)**	**Exome seq**	**RNA seq**	**microRNA seq**
					**29**	**26**	**27**
**1**	Saliva	M		56	1		
	NS		Left medial neck		1	1	1
	AK		Left dorsal forearm		1		1
**2**	NS	M	Left anterior shoulder	78	1	1	1
	AK		Left temple		1	1	1
	SCC		Left anterior shoulder		1	1	1
**3**	NS	M	Left infraorbital cheek	70	1	1	1
	AK		Right dorsal forearm		1	1	1
	SCC		Left infraorbital cheek		1	1	1
**4**	Saliva	F		60	1		
	NS		Left ulnar forearm		1	1	1
	AK1		Right anterior shoulder			1	1
	AK2		Left chest sternum		1	1	1
	SCC1		Left ulnar forearm		1	1	1
	SCC2		Left dorsal hand		1	1	1
**5**	Saliva	M		75	1		
	NS		Crown of scalp		1	1	1
	AK		Scalp		1	1	1
	SCC		Crown of scalp		1	1	1
**6**	Saliva	M		82	1		
	NS		Left sternocleidomastoid		1	1	1
	AK		Left zygomatic arch			1	1
	SCC		Left sternocleidomastoid		1	1	1
**8**	Saliva	M		83	1		
	NS		Right temple		1	1	1
	AK		Right temple			1	1
	SCC		Right temple		1	1	1
**10**	Saliva	M		79	1		
	NS		Left temporal hairline		1	1	1
	AK		Right scalp		1	1	1
	SCC		Left temporal hairline			1	1
**12**	Saliva	F		74	1		
	AK		Right knee		1	1	1
	SCC		Right pretibia			1	1

AK, actinic keratosis; NS, normal skin; SCC, squamous cell carcinoma.

**Table 2:** Site-specific mutational overlap.

**PT**	**NS Total**	**AK Total**	**SCC Total**	**NS/AK**	**AK/SCC**	**NS/SCC**	**NS/AK/SCC**	**SCC1 versus SCC2**	**NS/AK/SCC1**	**NS/AK/SCC2**	**NS/AK/SCC1/SCC2**
**1**	1,264	449	—	2	—	—	—	—	—	—	—
**2**	—	1,631	1,332	—	4	—	—	—	—	—	—
**3**	—	1,555	9,156	—	6	—	—	—	—	—	—
**4**	257	1,056	385	0	1	0	—	—	1	—	1
**4 (SCC2)**	—	—	578	—	0	1	—	195	—	0	1
**5**	24	1,447	4,924	1	6	0	1	—	—	—	—
**6**	347	—	3,614	—	—	6	—	—	—	—	—
**8**	319	—	503	—	—	2	—	—	—	—	—
**10**	23	1,873	—	0	—	—	—	—	—	—	—
**12**	—	290	—	—	—	—	—	—	—	—	—
**Average**				**0.75**	**3.4**	**1.8**					

AK, actinic keratosis; cuSCC, cutaneous squamous cell carcinoma; NS, normal skin.

The overlap of site-specific variants in NS, AK and cuSCC shows that the greatest amount of overlap between AK and cuSCC. These average 3.4 between AK and cuSCC, 0.75 between NS and AK, and 1.8 between NS and cuSCC. The most overlap occurred between two SCCs from patient 4 (195), which were from two distinct lesions in close physical proximity, suggesting that they may have arisen at least partially from a common clone.

## References

[b1] Rosen T. & Lebwohl M. G. Prevalence and awareness of actinic keratosis: barriers and opportunities. J. Am. Acad. Dermatol. 68, S2–S9 (2013). 23228302 10.1016/j.jaad.2012.09.052

[b2] Warino L. . Frequency and cost of actinic keratosis treatment. Dermatol. Surg. 32, 1045–1049 (2006). 16918567 10.1111/j.1524-4725.2006.32228.x

[b3] Criscione V. D. . Actinic keratoses: natural history and risk of malignant transformation in the Veterans Affairs Topical Tretinoin Chemoprevention Trial. Cancer 115, 2523–2530 (2009). 19382202 10.1002/cncr.24284

[b4] Karia P. S., Han J. & Schmults C. D. Cutaneous squamous cell carcinoma: estimated incidence of disease, nodal metastasis, and deaths from disease in the United States, 2012. J. Am. Acad. Dermatol. 68, 957–966 (2013). 23375456 10.1016/j.jaad.2012.11.037

[b5] Neidecker M. V., Davis-Ajami M. L., Balkrishnan R. & Feldman S. R. Pharmacoeconomic considerations in treating actinic keratosis. Pharmacoeconomics 27, 451–464 (2009). 19640009 10.2165/00019053-200927060-00002

[b6] Zaravinos A., Kanellou P. & Spandidos D. A. Viral DNA detection and RAS mutations in actinic keratosis and nonmelanoma skin cancers. Br. J. Dermatol. 162, 325–331 (2010). 19849697 10.1111/j.1365-2133.2009.09480.x

[b7] Kanellou P. . Genomic instability, mutations and expression analysis of the tumour suppressor genes p14(ARF), p15(INK4b), p16(INK4a) and p53 in actinic keratosis. Cancer. Lett. 264, 145–161 (2008). 18331779 10.1016/j.canlet.2008.01.042

[b8] Pacifico A. . Loss of CDKN2A and p14ARF expression occurs frequently in human nonmelanoma skin cancers. Br. J. Dermatol. 158, 291–297 (2008). 18070208 10.1111/j.1365-2133.2007.08360.x

[b9] Rehman I., Takata M., Wu Y. Y. & Rees J. L. Genetic change in actinic keratoses. Oncogene 12, 2483–2490 (1996). 8700506

[b10] Toll A. . Epidermal growth factor receptor gene numerical aberrations are frequent events in actinic keratoses and invasive cutaneous squamous cell carcinomas. Exp. Dermatol. 19, 151–153 (2010). 20156290 10.1111/j.1600-0625.2009.01028.x

[b11] Toll A. . MYC gene numerical aberrations in actinic keratosis and cutaneous squamous cell carcinoma. Br. J. Dermatol. 161, 1112–1118 (2009). 19673870 10.1111/j.1365-2133.2009.09351.x

[b12] Salgado R. . CKS1B amplification is a frequent event in cutaneous squamous cell carcinoma with aggressive clinical behaviour. Genes Chromosomes Cancer 49, 1054–1061 (2010). 20737481 10.1002/gcc.20814

[b13] Sekulic A. . Loss of inositol polyphosphate 5-phosphatase is an early event in development of cutaneous squamous cell carcinoma. Cancer Prev. Res. (Phila.) 3, 1277–1283 (2010). 20876729 10.1158/1940-6207.CAPR-10-0058 PMC2955780

[b14] Padilla R. S., Sebastian S., Jiang Z., Nindl I. & Larson R. Gene expression patterns of normal human skin, actinic keratosis, and squamous cell carcinoma: a spectrum of disease progression. Arch. Dermatol. 146, 288–293 (2010). 20231500 10.1001/archdermatol.2009.378

[b15] Nindl I. . Identification of differentially expressed genes in cutaneous squamous cell carcinoma by microarray expression profiling. Mol. Cancer 5, 30 (2006). 16893473 10.1186/1476-4598-5-30 PMC1569867

[b16] Hameetman L. . Molecular profiling of cutaneous squamous cell carcinomas and actinic keratoses from organ transplant recipients. BMC Cancer 13, 58 (2013). 23379751 10.1186/1471-2407-13-58 PMC3570297

[b17] Dooley T. P., Reddy S. P., Wilborn T. W. & Davis R. L. Biomarkers of human cutaneous squamous cell carcinoma from tissues and cell lines identified by DNA microarrays and qRT-PCR. Biochem. Biophys. Res. Commun. 306, 1026–1036 (2003). 12821146 10.1016/s0006-291x(03)01099-4

[b18] Haider A. S. . Genomic analysis defines a cancer-specific gene expression signature for human squamous cell carcinoma and distinguishes malignant hyperproliferation from benign hyperplasia. J. Invest. Dermatol. 126, 869–881 (2006). 16470182 10.1038/sj.jid.5700157

[b19] Kathpalia V. P. . Genome-wide transcriptional profiling in human squamous cell carcinoma of the skin identifies unique tumor-associated signatures. J. Dermatol. 33, 309–318 (2006). 16700662 10.1111/j.1346-8138.2006.00075.x

[b20] Ra S. H., Li X. & Binder S. Molecular discrimination of cutaneous squamous cell carcinoma from actinic keratosis and normal skin. Mod. Pathol. 24, 963–973 (2011). 21743436 10.1038/modpathol.2011.39

[b21] Hudson L. G. . Microarray analysis of cutaneous squamous cell carcinomas reveals enhanced expression of epidermal differentiation complex genes. Mol. Carcinog. 49, 619–629 (2010). 20564339 10.1002/mc.20636 PMC3626076

[b22] Harbig J., Sprinkle R. & Enkemann S. A. A sequence-based identification of the genes detected by probesets on the Affymetrix U133 plus 2.0 array. Nucleic Acids Res. 33, e31 (2005). 15722477 10.1093/nar/gni027 PMC549426

[b23] Benavides F., Oberyszyn T. M., VanBuskirk A. M., Reeve V. E. & Kusewitt D. F. The hairless mouse in skin research. J. Dermatol. Sci. 53, 10–18 (2009). 18938063 10.1016/j.jdermsci.2008.08.012 PMC2646590

[b24] Vin H. . BRAF inhibitors suppress apoptosis through off-target inhibition of JNK signaling. Elife 2, e00969 (2013). 24192036 10.7554/eLife.00969 PMC3814616

[b25] van Kranen H. J. . Frequent p53 alterations but low incidence of ras mutations in UV-B-induced skin tumors of hairless mice. Carcinogenesis 16, 1141–1147 (1995). 7767977 10.1093/carcin/16.5.1141

[b26] Khan S. G. . Mutations in ras oncogenes: rare events in ultraviolet B radiation-induced mouse skin tumorigenesis. Mol. Carcinog. 15, 96–103 (1996). 8599584 10.1002/(SICI)1098-2744(199602)15:2<96::AID-MC2>3.0.CO;2-P

[b27] Soufir N. . INK4a-ARF mutations in skin carcinomas from UV irradiated hairless mice. Mol. Carcinog. 39, 195–198 (2004). 15057871 10.1002/mc.20004

[b28] Ashton K. J., Weinstein S. R., Maguire D. J. & Griffiths L. R. Chromosomal aberrations in squamous cell carcinoma and solar keratoses revealed by comparative genomic hybridization. Arch. Dermatol. 139, 876–882 (2003). 12873882 10.1001/archderm.139.7.876

[b29] Popp S. . Genetic characterization of a human skin carcinoma progression model: from primary tumor to metastasis. J. Invest. Dermatol. 115, 1095–1103 (2000). 11121147 10.1046/j.1523-1747.2000.00173.x

[b30] Dworkin A. M. . Chromosomal aberrations in UVB-induced tumors of immunosuppressed mice. Genes Chromosomes Cancer 48, 490–501 (2009). 19296524 10.1002/gcc.20657 PMC2739622

[b31] Rundhaug J. E. . SAGE profiling of UV-induced mouse skin squamous cell carcinomas, comparison with acute UV irradiation effects. Mol. Carcinog. 42, 40–52 (2005). 15547921 10.1002/mc.20064

[b32] South A. P. . NOTCH1 mutations occur early during cutaneous squamous cell carcinogenesis. J. Invest. Dermatol. 134, 2630–2638 (2014). 24662767 10.1038/jid.2014.154 PMC4753672

[b33] Wang N. J. . Loss-of-function mutations in Notch receptors in cutaneous and lung squamous cell carcinoma. Proc. Natl Acad. Sci. USA 108, 17761–17766 (2011). 22006338 10.1073/pnas.1114669108 PMC3203814

[b34] Pickering C. R. . Mutational landscape of aggressive cutaneous squamous cell carcinoma. Clin. Cancer Res. 20, 6582–6592 (2014). 25303977 10.1158/1078-0432.CCR-14-1768 PMC4367811

[b35] Li Y. Y. . Genomic analysis of metastatic cutaneous squamous cell carcinoma. Clin. Cancer Res. 21, 1447–1456 (2015). 25589618 10.1158/1078-0432.CCR-14-1773 PMC4359951

[b36] Martincorena I. . Tumor evolution. High burden and pervasive positive selection of somatic mutations in normal human skin. Science 348, 880–886 (2015). 25999502 10.1126/science.aaa6806 PMC4471149

[b37] Jonason A. S. . Frequent clones of p53-mutated keratinocytes in normal human skin. Proc. Natl Acad. Sci. USA 93, 14025–14029 (1996). 8943054 10.1073/pnas.93.24.14025 PMC19488

[b38] Brash D. E. UV signature mutations. Photochem. Photobiol. 91, 15–26 (2015). 25354245 10.1111/php.12377 PMC4294947

[b39] Covington K. R., Shinbrot E. & Wheeler D. A. Mutation signatures reveal biological processes in human cancer, bioRxiv. http://dx.doi.org/10.1101/036541 (2016).

[b40] Lee C. S. . Recurrent point mutations in the kinetochore gene KNSTRN in cutaneous squamous cell carcinoma. Nat. Genet. 46, 1060–1062 (2014). 25194279 10.1038/ng.3091 PMC4324615

[b41] Ziegler A. . Mutation hotspots due to sunlight in the p53 gene of nonmelanoma skin cancers. Proc. Natl Acad. Sci. USA 90, 4216–4220 (1993). 8483937 10.1073/pnas.90.9.4216 PMC46477

[b42] Carter S. L., Eklund A. C., Kohane I. S., Harris L. N. & Szallasi Z. A signature of chromosomal instability inferred from gene expression profiles predicts clinical outcome in multiple human cancers. Nat. Genet. 38, 1043–1048 (2006). 16921376 10.1038/ng1861

[b43] Kar A. & Gutierrez-Hartmann A. Molecular mechanisms of ETS transcription factor-mediated tumorigenesis. Crit. Rev. Biochem. Mol. Biol. 48, 522–543 (2013). 24066765 10.3109/10409238.2013.838202 PMC4086824

[b44] Einspahr J. G. . Functional protein pathway activation mapping of the progression of normal skin to squamous cell carcinoma. Cancer Prev. Res. (Phila.) 5, 403–413 (2012). 22389437 10.1158/1940-6207.CAPR-11-0427 PMC3297971

[b45] Su F. . RAS mutations in cutaneous squamous-cell carcinomas in patients treated with BRAF inhibitors. N. Engl. J. Med. 366, 207–215 (2012). 22256804 10.1056/NEJMoa1105358 PMC3724537

[b46] Kumar A. P. & Butler A. P. Enhanced Sp1 DNA-binding activity in murine keratinocyte cell lines and epidermal tumors. Cancer Lett. 137, 159–165 (1999). 10374837 10.1016/s0304-3835(98)00351-6

[b47] Miao Q. . Tcf3 promotes cell migration and wound repair through regulation of lipocalin 2. Nat. Commun. 5, 4088 (2014). 24909826 10.1038/ncomms5088 PMC4052366

[b48] Bhatia N. & Spiegelman V. S. Activation of Wnt/beta-catenin/Tcf signaling in mouse skin carcinogenesis. Mol. Carcinog. 42, 213–221 (2005). 15765534 10.1002/mc.20077

[b49] Mammucari C. . Integration of Notch 1 and calcineurin/NFAT signaling pathways in keratinocyte growth and differentiation control. Dev. Cell. 8, 665–676 (2005). 15866158 10.1016/j.devcel.2005.02.016

[b50] Eckert R. L. . AP1 transcription factors in epidermal differentiation and skin cancer. J. Skin Cancer 2013, 537028 (2013). 23762562 10.1155/2013/537028 PMC3676924

[b51] Wang Q. S., Kong P. Z., Li X. Q., Yang F. & Feng Y. M. FOXF2 deficiency promotes epithelial-mesenchymal transition and metastasis of basal-like breast cancer. Breast Cancer Res. 17, 30 (2015). 25848863 10.1186/s13058-015-0531-1 PMC4361145

[b52] Ooi A. T. . Molecular profiling of premalignant lesions in lung squamous cell carcinomas identifies mechanisms involved in stepwise carcinogenesis. Cancer Prev. Res. (Phila) 7, 487–495 (2014). 24618292 10.1158/1940-6207.CAPR-13-0372 PMC4059064

[b53] Chang J. T. & Nevins J. R. GATHER: a systems approach to interpreting genomic signatures. Bioinformatics 22, 2926–2933 (2006). 17000751 10.1093/bioinformatics/btl483

[b54] Gunaratne P. H., Coarfa C., Soibam B. & Tandon A. miRNA data analysis: next-gen sequencing. Methods Mol. Biol. 822, 273–288 (2012). 22144206 10.1007/978-1-61779-427-8_19

[b55] Bruegger C. . MicroRNA expression differs in cutaneous squamous cell carcinomas and healthy skin of immunocompetent individuals. Exp. Dermatol. 22, 426–428 (2013). 23711067 10.1111/exd.12153

[b56] Dziunycz P. . Squamous cell carcinoma of the skin shows a distinct microRNA profile modulated by UV radiation. J. Invest. Dermatol. 130, 2686–2689 (2010). 20574436 10.1038/jid.2010.169

[b57] Wang A. . MicroRNA-31 is overexpressed in cutaneous squamous cell carcinoma and regulates cell motility and colony formation ability of tumor cells. PLoS One 9, e103206 (2014). 25068518 10.1371/journal.pone.0103206 PMC4113372

[b58] Akilesh S. . Arhgap24 inactivates Rac1 in mouse podocytes, and a mutant form is associated with familial focal segmental glomerulosclerosis. J. Clin. Invest. 121, 4127–4137 (2011). 21911940 10.1172/JCI46458 PMC3195463

[b59] Wang W. . PTPN14 is required for the density-dependent control of YAP1. Genes Dev. 26, 1959–1971 (2012). 22948661 10.1101/gad.192955.112 PMC3435498

[b60] Kasem K. . JK1 (FAM134B) represses cell migration in colon cancer: a functional study of a novel gene. Exp. Mol. Pathol. 97, 99–104 (2014). 24927874 10.1016/j.yexmp.2014.06.002

[b61] Stransky N. . The mutational landscape of head and neck squamous cell carcinoma. Science 333, 1157–1160 (2011). 21798893 10.1126/science.1208130 PMC3415217

[b62] Agrawal N. . Exome sequencing of head and neck squamous cell carcinoma reveals inactivating mutations in NOTCH1. Science 333, 1154–1157 (2011). 21798897 10.1126/science.1206923 PMC3162986

[b63] Cancer Genome Atlas, N. Comprehensive genomic characterization of head and neck squamous cell carcinomas. Nature 517, 576–582 (2015). 25631445 10.1038/nature14129 PMC4311405

[b64] Hammerman P. S. . Comprehensive genomic characterization of squamous cell lung cancers. Nature 489, 519–525 (2012). 22960745 10.1038/nature11404 PMC3466113

[b65] Lin D. C. . Genomic and molecular characterization of esophageal squamous cell carcinoma. Nat. Genet. 46, 467–473 (2014). 24686850 10.1038/ng.2935 PMC4070589

[b66] Bass A. J. . SOX2 is an amplified lineage-survival oncogene in lung and esophageal squamous cell carcinomas. Nat. Genet. 41, 1238–1242 (2009). 19801978 10.1038/ng.465 PMC2783775

[b67] Arron S. T., Ruby J. G., Dybbro E., Ganem D. & Derisi J. L. Transcriptome sequencing demonstrates that human papillomavirus is not active in cutaneous squamous cell carcinoma. J. Invest. Dermatol. 131, 1745–1753 (2011). 21490616 10.1038/jid.2011.91 PMC3136639

[b68] Ross-Innes C. S. . Whole-genome sequencing provides new insights into the clonal architecture of Barrett's esophagus and esophageal adenocarcinoma. Nat. Genet. 47, 1038–1046 (2015). 26192915 10.1038/ng.3357 PMC4556068

[b69] Stachler M. D. . Paired exome analysis of Barrett's esophagus and adenocarcinoma. Nat. Genet. 47, 1047–1055 (2015). 26192918 10.1038/ng.3343 PMC4552571

[b70] Nassar D., Latil M., Boeckx B., Lambrechts D. & Blanpain C. Genomic landscape of carcinogen-induced and genetically induced mouse skin squamous cell carcinoma. Nat. Med. 21, 946–954 (2015). 26168291 10.1038/nm.3878

[b71] Nghiem D. X., Walterscheid J. P., Kazimi N. & Ullrich S. E. Ultraviolet radiation-induced immunosuppression of delayed-type hypersensitivity in mice. Methods 28, 25–33 (2002). 12231185 10.1016/s1046-2023(02)00207-4

[b72] Alexandrov L. B. . Signatures of mutational processes in human cancer. Nature 500, 415–421 (2013). 23945592 10.1038/nature12477 PMC3776390

[b73] Lee W. P. . MOSAIK: a hash-based algorithm for accurate next-generation sequencing short-read mapping. PLoS One 9, e90581 (2014). 24599324 10.1371/journal.pone.0090581 PMC3944147

[b74] Love M. I., Huber W. & Anders S. Moderated estimation of fold change and dispersion for RNA-seq data with DESeq2. Genome Biol. 15, 550 (2014). 25516281 10.1186/s13059-014-0550-8 PMC4302049

[b75] Chen Y. . VirusSeq: software to identify viruses and their integration sites using next-generation sequencing of human cancer tissue. Bioinformatics. 29, 266–267 (2013). 23162058 10.1093/bioinformatics/bts665 PMC3546792

[b76] Kozomara A. & Griffiths-Jones S. miRBase: annotating high confidence microRNAs using deep sequencing data. Nucleic Acids Res. 42, D68–D73 (2014). 24275495 10.1093/nar/gkt1181 PMC3965103

[b77] Johnson W. E., Li C. & Rabinovic A. Adjusting batch effects in microarray expression data using empirical Bayes methods. Biostatistics 8, 118–127 (2007). 16632515 10.1093/biostatistics/kxj037

[b78] Agarwal V., Bell G. W., Nam J. W. & Bartel D. P. Predicting effective microRNA target sites in mammalian mRNAs. Elife 4, 1–38 doi:10.7554/eLife.05005 (2015). PMC4532895 26267216

[b79] Subramanian A. . Gene set enrichment analysis: a knowledge-based approach for interpreting genome-wide expression profiles. Proc. Natl Acad. Sci. USA 102, 15545–15550 (2005). 16199517 10.1073/pnas.0506580102 PMC1239896

[b80] Krzywinski M. . Circos: an information aesthetic for comparative genomics. Genome Res. 19, 1639–1645 (2009). 19541911 10.1101/gr.092759.109 PMC2752132

