# Supplementary material for: Cross-species identification of genomic drivers of squamous cell carcinoma development across preneoplastic intermediates
Source: Nat Commun. 2016 Aug 30;7:12601. doi: 10.1038/ncomms12601 (PMC5013636; doi:10.1038/ncomms12601)
Supplement: Supplementary Information — Supplementary Figures 1-8 and Supplementary Tables 1-2 [file ncomms12601-s1.pdf]

Supplementary Figures

Dinucleotide variant proportions

Change	CC>TT	GG>AA	Other
NS	48	39	17
AK	205	276	53
SCC	580	590	137

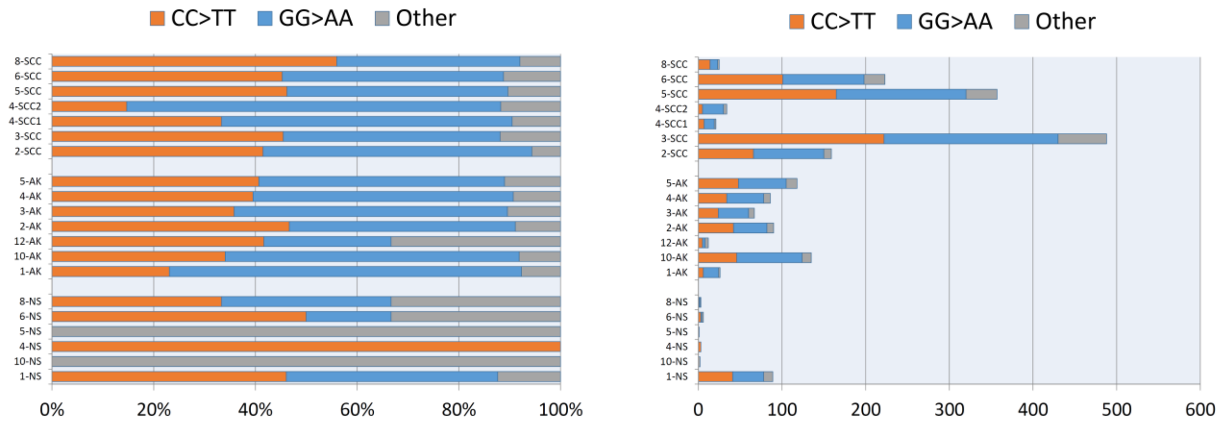

Supplementary Figure 1. Dinucleotide variant proportions. These are described and quantitated for each lesion type.

a

b

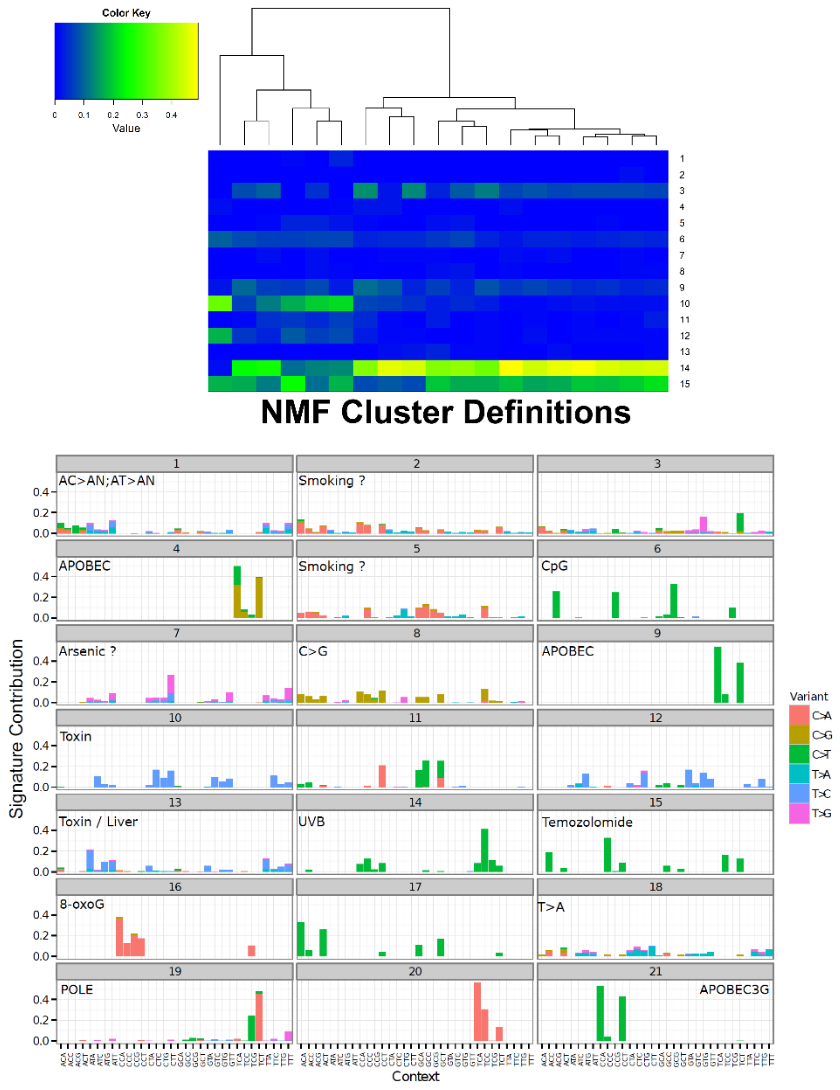

Supplementary Figure 2. Non-negative matrix factorization-derived orthogonal mutational profiles. (a) Heatmap of NMF-derived orthogonal mutational profiles derived from over 6,000 human cancers for the human exome samples. The scale bar refers to the proportion of mutations attributable to a given signature. The clustering confirms a strong enrichment for CpG-associated C→T transitions classically associated with UVB-exposure (signature 14), particularly for AK and cuSCC, with segregation of most of the NS samples away from AK and cuSCC, with the exception of 1-NS, which has the highest mutational load in that class. Two other profiles dominated by C→T transitions are significantly represented in the mutational data and relatively enriched in NS (signatures 10, 15), including one first described in the context of temozolamide exposure (signature 15). (b) Table of NMF Cluster Definitions. Our strategy employed non-smooth NMF, a variant which approximates the data using the basis and

coefficient matrices as above with the addition of a third smoothing matrix which serves to absorb noise within the data, thus driving the coefficient and basis matrices to increase sparseness. The resulting basis matrix generated k=21 signatures from a diverse set of over 6000 cancers[39], reducing correlations between signatures originally derived by Alexandrov et al.[72] thereby increasing orthogonality.

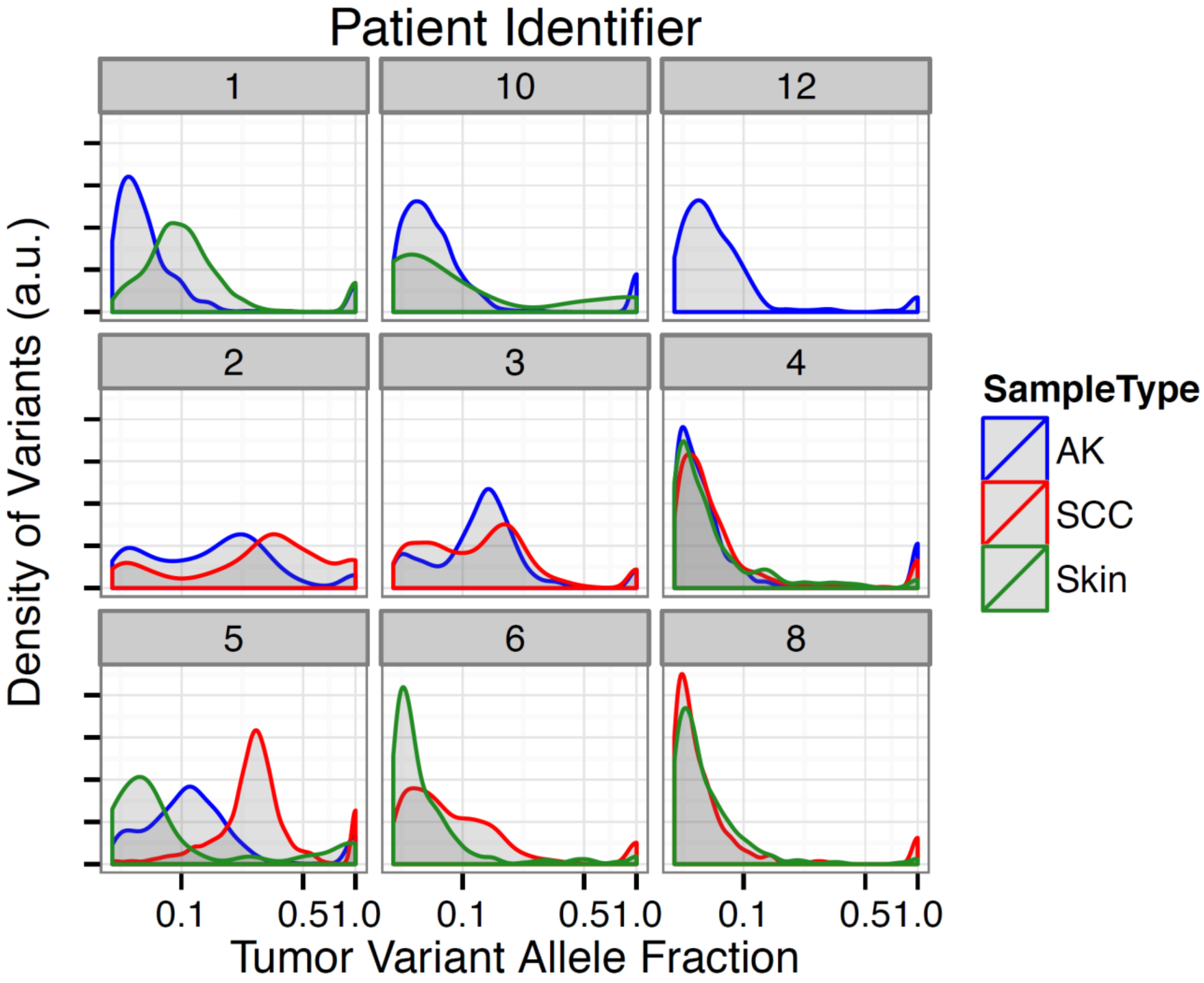

Supplementary Figure 3. Histograms of variant allele frequencies of samples grouped by patient.

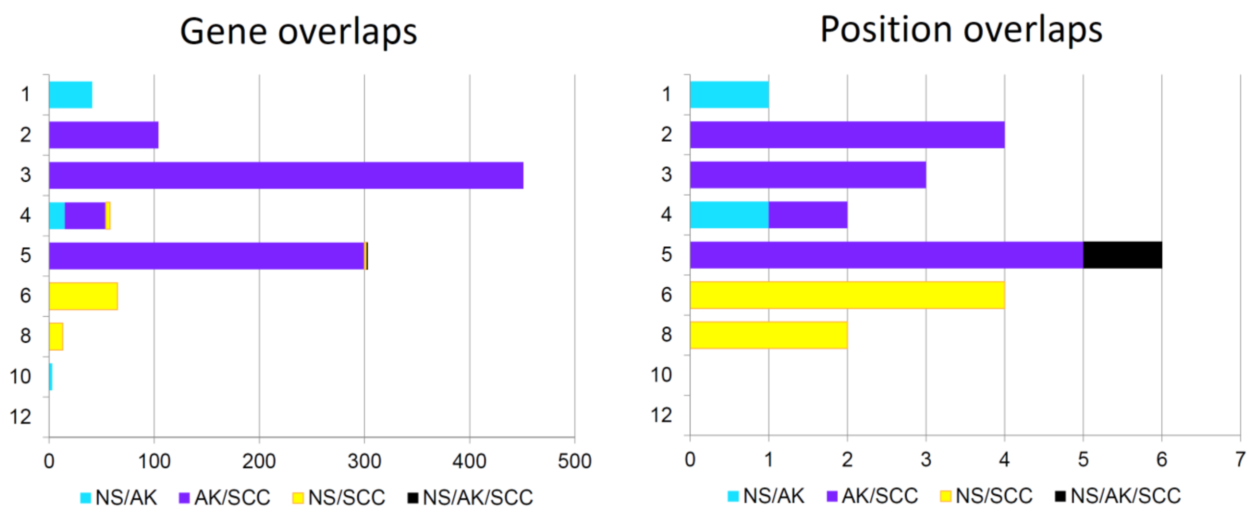

Supplementary Figure 4. Enumeration of overlaps between mutated genes and variant positions within mutated genes within samples. Overlaps in non-silent SNVs at the gene and position level are enumerated here.

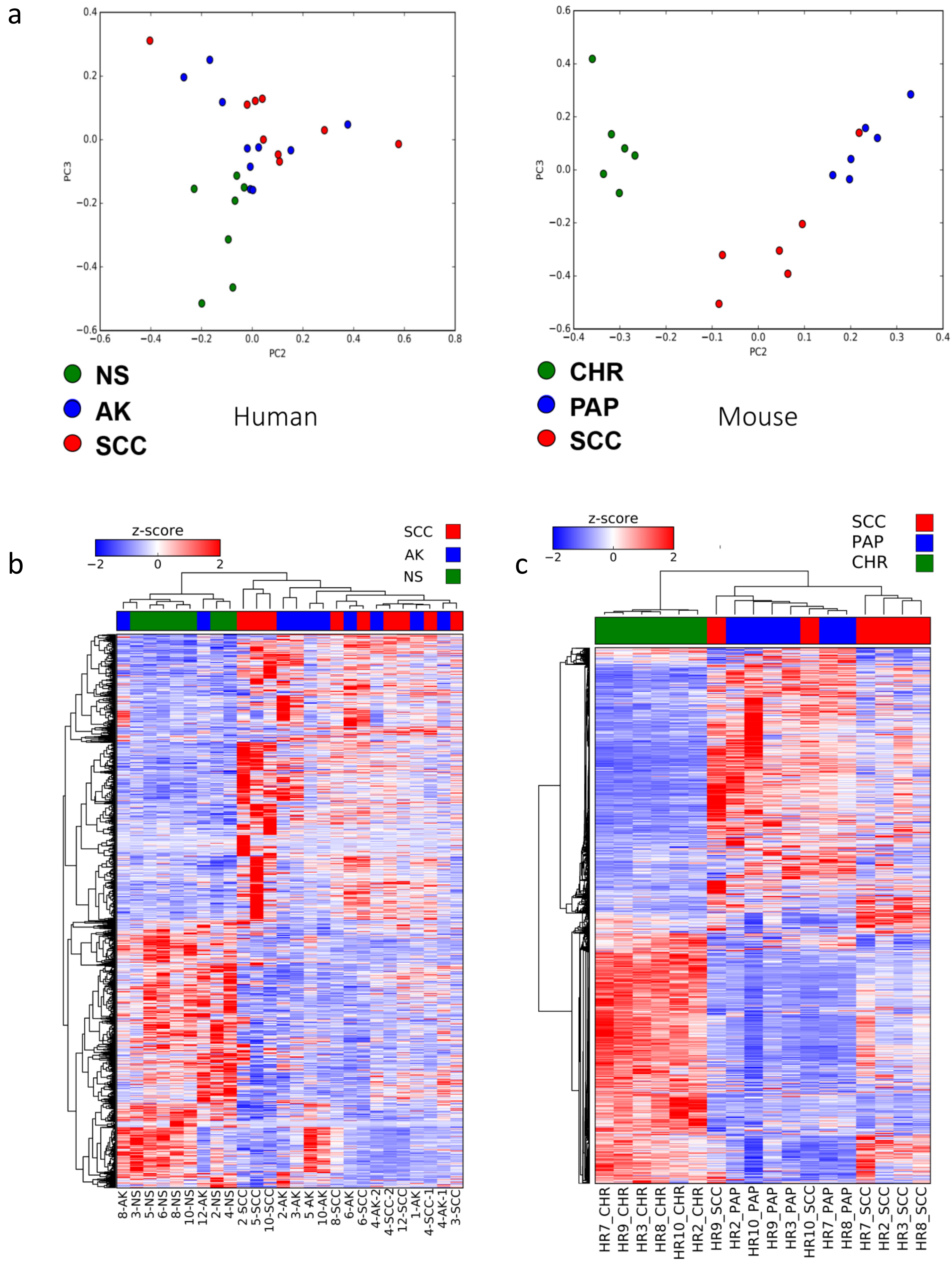
a
PC3
PC2
NS
AK
SCC
Human
CHR
PAP
SCC
Mouse
b
z-score
SCC
AK
NS
8-AK
3-NS
5-NS
6-NS
8-NS
10-NS
12-AK
2-NS
4-NS
2-SCC
5-SCC
10-SCC
2-AK
3-AK
5-AK
10-AK
8-SCC
6-AK
6-SCC
4-AK-2
4-SCC-2
12-SCC
1-AK
4-SCC-1
4-AK-1
3-SCC
c
z-score
SCC
PAP
CHR
HR7_CHR
HR9_CHR
HR3_CHR
HR8_CHR
HR10_CHR
HR2_CHR
HR9_SCC
HR2_PAP
HR10_PAP
HR9_PAP
HR3_PAP
HR10_SCC
HR7_PAP
HR8_PAP
HR7_SCC
HR2_SCC
HR3_SCC
HR8_SCC

Supplementary Figure 5. Analysis of mRNA expression in human and mouse lesions. (a) Principal component analysis of unsupervised mRNA expression. Using principal component analysis across three dimensions as an alternative means of organizing the transcriptional profiles, the results of the unsupervised clustering are largely recapitulated: cuSCC and AK/PAP segregate away from NS most prominently. (b) Clustering of recurrently differentially expressed human genes in at least 2 of 3 possible pairwise comparisons between NS, AK, and cuSCC shows that AKs cluster predominantly with cuSCC, consistent with the notion that they are largely transcriptomically more similar to cuSCC. (c) Clustering of recurrently differentially expressed mouse genes in at least 2 of 3 possible pairwise comparisons between CHR, PAP, and cuSCC shows that PAP cluster predominantly with cuSCC, consistent with the notion that they are largely transcriptomically more similar to cuSCC.

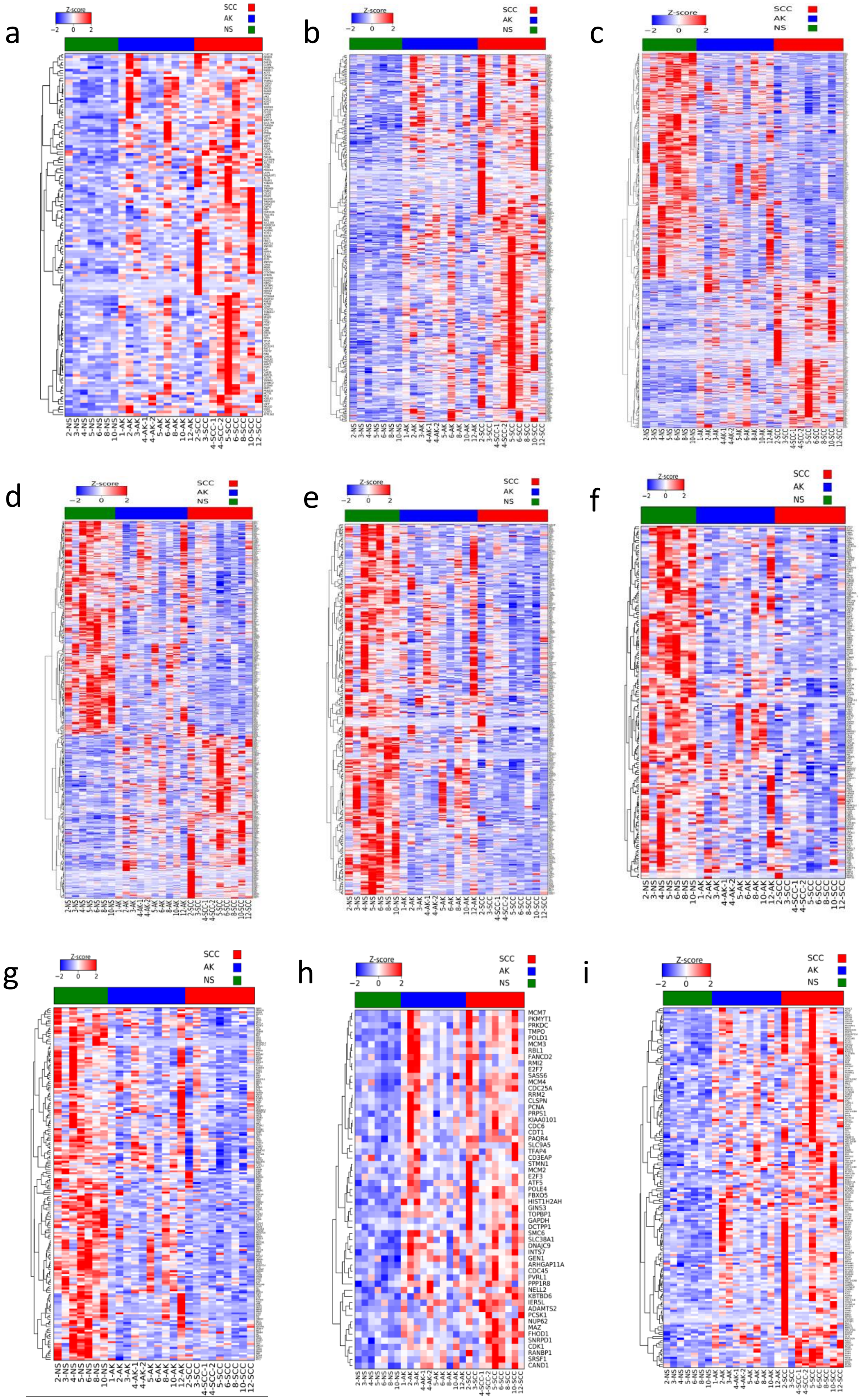
a
b
c
d
e
f
g
h
i
Z-score
-2 0 2
SCC
AK
NS
2-NS
3-NS
4-NS
5-NS
6-NS
8-NS
10-NS
1-AK
2-AK
3-AK
4-AK-1
4-AK-2
5-AK
6-AK
8-AK
10-AK
12-AK
2-SCC
3-SCC
4-SCC-1
4-SCC-2
5-SCC
6-SCC
8-SCC
10-SCC
12-SCC
MCM7
PKMYT1
PRKDC
TMPO
POLD1
MCM3
RBL1
FANCD2
RMI2
E2F7
SASS6
MCM4
CDC25A
RRM2
CLSPN
PCNA
PRPS1
KIAA0101
CDC6
CDT1
PAQR4
SLC9A5
TFAP4
CD3EAP
STMN1
MCM2
E2F3
ATF5
POLE4
FBXO5
HIST1H2AH
GINS3
TOPBP1
GAPDH
DCTPP1
SMC6
SLC38A1
DNAJC9
INTS7
GEN1
ARHGAP11A
CDC45
PVRL1
PPP1R8
NELL2
KBTBD6
IER5L
ADAMTS2
PCSK1
NUP62
MAZ
FHOD1
SNRPD1
CDK1
RANBP1
SRSF1
CAND1

Supplementary Figure 6. TRANSFAC based analysis of transcription factor regulators. (a) ETS2 targets and (b) SP1 target are upregulated across the continuum of samples. (c) TCF3 targets that are overall downregulated have greater significance across the continuum than those that are upregulated in the late stage transition (AK/PAP to cuSCC). (d) LEF1 targets also exhibit a similar mixed pattern, with one subset of target genes with significant downregulation early (NS/CHR to AK/PAP) and a distinct subset showing upregulation late (AK/PAP to cuSCC). The majority of NFAT targets (e) also exhibit downregulation early. (f) AP1 and (g) FREAC2 (FOXF2) targets are downregulated across the continuum of cuSCC development. Targets of (h) E2F and (i) NFY are significantly upregulated early (NS to AK/PAP) and these were also highlighted in the linear mixed-effects (LME) model of progression which are highlighted in red in panel (Fig. 5a).

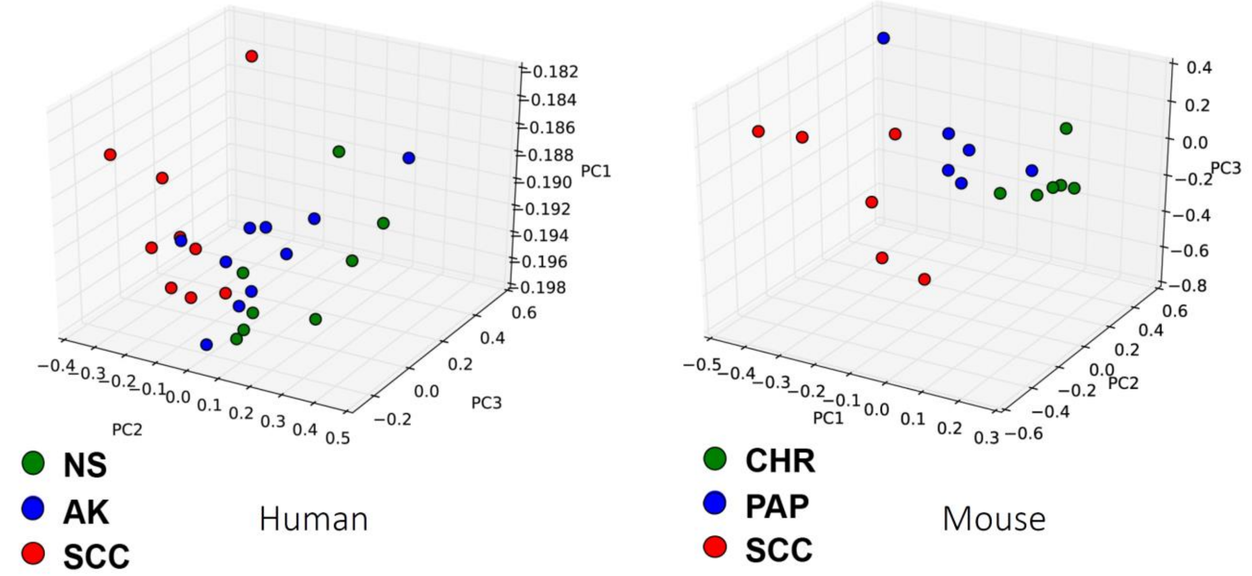

Supplementary Figure 7. Principal component analysis of unsupervised microRNA expression. Using principal component analysis across three dimensions as an alternative means of organizing the microRNA profiles, the results of the unsupervised clustering are largely recapitulated: while NS/CHR are very distinctly separated from cuSCC, segregation of AK/PAP as a distinct group is more clearly seen here as compared to the principal component analysis of mRNA profiles, particularly in mouse.

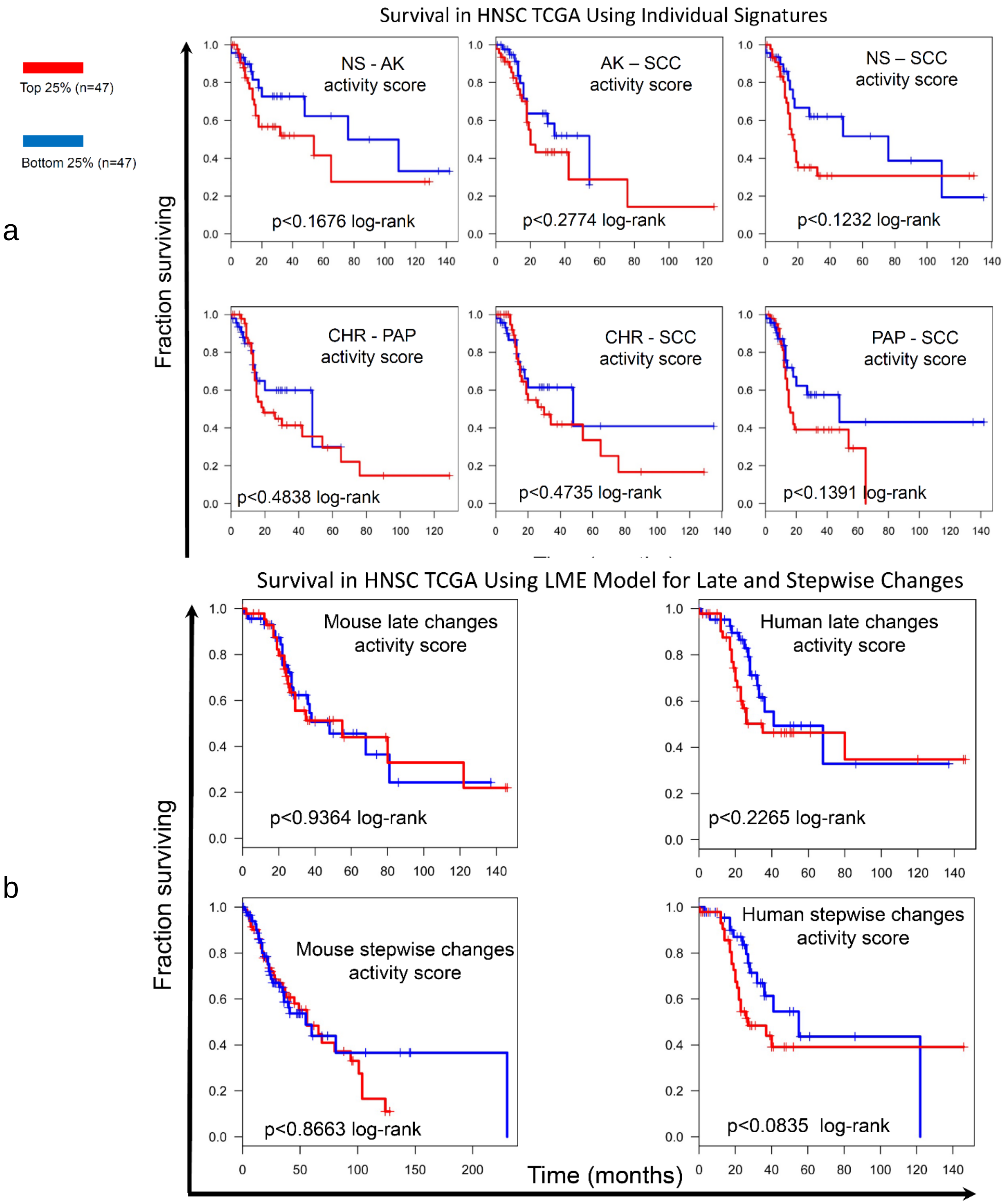

Supplementary Figure 8. Survival analysis for head & neck squamous cell carcinoma. (a) All of the individual signatures for all significant pairwise comparisons in human and mouse were probed for their ability to predict survival in *TP53*-mutant HNSCC in the top and bottom 25% of ranked signatures; none was significant. (b) The remaining late transition and stepwise (both

early and late) signatures from the LME model data were probed for their ability to predict survival in HNSCC; none was significant.

Supplementary Tables

Gene	#	Patients
GPR98	4	PT1,PT2,PT3,PT4
MYO7B	4	PT1,PT3,PT4,PT5
RELN	4	PT1,PT3,PT4,PT5
TP53	4	PT1,PT3,PT4,PT5
CRB1	3	PT2,PT3,PT6
FAT1	3	PT3,PT4,PT5
FSIP2	3	PT2,PT3,PT5
IGFN1	3	PT2,PT3,PT5
MLL3	3	PT2,PT5,PT6
RYR1	3	PT2,PT4,PT5
VWDE	3	PT2,PT3,PT5
ADAM29	2	PT3,PT4
ADGB	2	PT2,PT3
ANKRD31	2	PT3,PT6
ATP8A1	2	PT3,PT5
BTBD9	2	PT3,PT5
C16orf62	2	PT3,PT4
C2orf16	2	PT4,PT5
CCDC168	2	PT3,PT5
CHD8	2	PT1,PT4
CLTCL1	2	PT3,PT6
CMYA5	2	PT4,PT5
COL11A1	2	PT4,PT5
COL1A2	2	PT3,PT5
COL24A1	2	PT3,PT5
COL27A1	2	PT4,PT5
COL4A2	2	PT2,PT3
COL4A3	2	PT3,PT4
COL6A5	2	PT3,PT5
COL6A6	2	PT2,PT5
CSF3R	2	PT3,PT5
CXorf22	2	PT3,PT4
DDX60L	2	PT3,PT4
DENND4A	2	PT4,PT5
DGKH	2	PT4,PT5
DNHD1	2	PT2,PT5
DPP3	2	PT5,PT6
DYNC1I1	2	PT3,PT5
ENPP3	2	PT2,PT5
FAM135A	2	PT5,PT8
FER1L6	2	PT3,PT5
FGA	2	PT3,PT5
FREM3	2	PT3,PT5
FRY	2	PT3,PT5
GLDN	2	PT5,PT6
HMCN1	2	PT3,PT5
KIAA1324L	2	PT3,PT5
KIAA1549	2	PT2,PT3
LAMA1	2	PT3,PT4
LAMC3	2	PT3,PT6
LGI1	2	PT1,PT3
LRP2	2	PT3,PT5
MLL2	2	PT2,PT5
MYH2	2	PT3,PT5
MYH4	2	PT3,PT5
MYO18B	2	PT3,PT4
NAV3	2	PT2,PT5
NHSL2	2	PT3,PT5
NPAP1	2	PT3,PT5
PALM2-AKAP2	2	PT2,PT3
PAPPA2	2	PT3,PT5
POLR3A	2	PT4,PT5
PRB4	2	PT1,PT5
PRDM9	2	PT4,PT5
PREX1	2	PT2,PT4
PRKD1	2	PT3,PT4
PRR16	2	PT3,PT5
PRRC2A	2	PT5,PT6
PRUNE2	2	PT2,PT3
PTPRQ	2	PT3,PT5
ROS1	2	PT3,PT5
RP1	2	PT3,PT5
SCAND3	2	PT3,PT6
SLC12A3	2	PT2,PT5
SLC26A7	2	PT4,PT6
SPAG17	2	PT3,PT5
STARD9	2	PT1,PT3
TCF20	2	PT5,PT6
THSD7A	2	PT2,PT4
TMEM131	2	PT2,PT5
TRDN	2	PT1,PT3
TRIM58	2	PT3,PT4
TRIM66	2	PT2,PT4
TTC40	2	PT3,PT6
USH2A	2	PT2,PT6
VWF	2	PT3,PT5
WBP2NL	2	PT3,PT4
WBSCR17	2	PT3,PT5
WDR87	2	PT3,PT4
XIRP2	2	PT2,PT3
ZFHX4	2	PT2,PT3

Removed as common false positive genes:

Gene	#	Patients
TTN	7	PT1,PT2,PT3,PT4,PT5,PT6,PT8
MUC16	6	PT1,PT2,PT3,PT4,PT5,PT6
CSMD3	3	PT2,PT3,PT5
DNAH6	3	PT2,PT3,PT6
DNAH7	3	PT3,PT5,PT6
HYDIN	3	PT3,PT5,PT6
LRP1B	3	PT1,PT3,PT5
MUC4	3	PT2,PT3,PT5
SYNE1	3	PT3,PT4,PT5
ADAMTS20	2	PT2,PT4
CSMD1	2	PT3,PT5
CTTNBP2	2	PT5,PT6
DNAH10	2	PT1,PT5
DNAH14	2	PT3,PT5
DNAH5	2	PT2,PT4
DNAH8	2	PT3,PT5
KALRN	2	PT3,PT5
OR4K17	2	PT3,PT5
PCLO	2	PT1,PT5

Supplementary Table 1. Table of overlapping mutated genes across patients.

MOUSE

Top Canonical Pathways	
Name	p-value
Mitotic Roles of Polo-Like Kinase	1.92E-09
Cell Cycle Control of Chromosomal Replication	3.15E-09
Cell Cycle: G2/M DNA Damage Checkpoint Regulation	5.77E-06
Hereditary Breast Cancer Signaling	7.97E-06
Role of BRCA1 in DNA Damage Response	4.48E-05

Top Upstream Regulators	
Upstream Regulator	p-value of overlap
E2F4	1.00E-31
RABL6	2.58E-25
ERBB2	1.42E-18
TP53	1.16E-15
E2F1	1.99E-15

HUMAN

Top Canonical Pathways	
Name	p-value
Cell Cycle Control of Chromosomal Replication	3.19E-09
Mitotic Roles of Polo-Like Kinase	1.42E-08
Hereditary Breast Cancer Signaling	2.85E-05
Cell Cycle: G2/M DNA Damage Checkpoint Regulation	3.45E-05
Role of BRCA1 in DNA Damage Response	4.53E-05

Top Upstream Regulators	
Upstream Regulator	p-value of overlap
E2F4	1.06E-31
RABL6	5.21E-24
ERBB2	9.64E-18
TP53	4.02E-15
CDK4	4.09E-15

Supplementary Table 2. Ingenuity Pathway Analysis of differentially expressed genes in the early (NS/CHR to AK/PAP) transition. Using the significantly differentially expressed genes in the early transition from NS/CHR to AK/PAP in the linear mixed effects model, IPA analysis was conducted separately for human and mouse genes. There is striking similarity in the key canonical pathways and upstream regulators identified.
